# Vitamin D and Particulate Matter–Associated Immune Responses in People Living with HIV: A Randomized Trial with Exploratory DNA Methylation Analyses

**DOI:** 10.3390/nu18213710

**Published:** 2026-11-09

**Authors:** Huimin Ben, Yuqian Hua, Zixuan Ping, Shouxin Peng, Yuxuan Tan, Xiuli Zhao, Zhihao Xie, Xiaoxia Liu, Wei Liang

**Affiliations:** 1 School of Public Health, Faculty of Medicine, Yangzhou University, 136# Jiangyang Middle Road, Yangzhou 225009, China; 2 Central China Subcenter of National Center for Cardiovascular Diseases, 1# Fuwai Avenue, Zhengzhou 450046, China; 3 Department of Global Health, School of Public Health, Wuhan University, 115# Donghu Road, Wuhan 430071, China; 4 Zhenjiang Center for Disease Control and Prevention, 9# Huangshan South Road, Zhenjiang 212000, China

**Keywords:** atmospheric particulate matter, immune function, vitamin D, HIV, DNA methylation

## Abstract

Background: HIV-infected individuals with compromised immune function are particularly vulnerable to atmospheric particulate matter (PM). While Vitamin D is known to modulate inflammation and immune function, its effect on PM-induced immune impairment in HIV-infected individuals remains unclear. Methods: This randomized, double-blind, placebo-controlled trial in Wuhan, China, investigated Vitamin D’s protective effects. In total, 80 participants received daily Vitamin D or a placebo and were followed up every 28 days across four visits. PM exposure was assessed 72 h before each follow-up, with immune cell counts, immunomodulatory factors, and whole-genome DNA methylation profiles measured. Linear mixed-effects models were used to assess the association between PM exposure and cell counts, and to identify CpG sites associated with Vitamin D. Mediating models further evaluated the role of these CpG sites in linking Vitamin D intervention to cytokine changes. Results: From March to July, 2023, 75 participants received 271 follow-ups. Vitamin D supplementation significantly attenuated the negative associations between PM exposure and CD4+T cell and CD19+B cell counts. Compared with the placebo group, the estimated PM-associated changes in CD4+T cell counts differed by 3.37%, 2.21%, and 1.96% per 10 µg/m^3^ increase in PM_1_, PM_2.5_, and PM_10_ concentrations, respectively, while the corresponding differences for CD19+B cell counts were 4.71%, 2.98%, and 2.65%, respectively. Significant methylation changes were observed at 173 CpG sites related to Vitamin D. Of these, 25 sites—including cg27066321 (*PEX14*), cg08500112 (*PDE4D*), and cg25882580 (*CACNG6*)—were identified as potential mediators of the effects of vitamin D supplementation on TLR4, IL-6, and TNF-α levels, with mediation proportions ranging from 27.98% to 78.78%. Conclusions: Vitamin D supplementation may attenuate PM-associated immune alterations in people living with HIV, with DNA methylation potentially contributing to the underlying mechanisms.

## 1. Introduction

AIDS remains a major global health burden, with approximately 37.7 million individuals living with HIV by 2020 [1]. HIV-infected individuals have compromised immune functions, leading to higher risks of co-infections, cancers, autoimmune diseases, and death [2,3]. Besides direct viral effects, immune competence in HIV-infected individuals is influenced by age, nutrition, comorbidities, treatment, and environmental exposures [4]. Among these, atmospheric particulate matter (PM) has emerged as a critical environmental factor linked to impaired immune responses and worsened clinical outcomes in this population [5]. Identifying effective interventions to alleviate PM-induced immune dysfunction in HIV-infected individuals is crucial for improving their health outcomes.

Vitamin D plays a critical role in regulating immune cell function, inhibiting inflammatory responses, and modulating oxidative stress. Deficiency in Vitamin D is linked to heightened susceptibility to infections, systemic inflammation, and chronic diseases [6,7]. Conversely, sufficient Vitamin D helps mitigate excessive inflammation and oxidative stress, maintaining immune homeostasis [8]. Inflammation and oxidative stress have been identified as potential biological mechanisms through which PM exposure impairs immune function [9]. Kewcharoenwong et al. demonstrated that PM exposure triggers the inflammatory cytokine IL-8 production by neutrophils, a process that Vitamin D supplementation can mitigate [10]. Additionally, Vitamin D has been shown to inhibit IL-6 production by bronchial epithelial cells, regulate oxidative stress pathways, and alleviate PM_2.5_-induced inflammatory responses, thereby reducing cellular damage [11]. Despite these cellular-level findings, the efficacy of Vitamin D supplementation in alleviating PM-induced immune impairment in HIV-infected individuals, particularly those with Vitamin D deficiency, remains unclear.

Vitamin D modulates DNA methylation by forming a complex with the Vitamin D receptor, which influences the expression and activity of DNA methyltransferases [12]. Altered DNA methylation has been implicated in immune dysfunction, particularly in the differentiation of immune cells (e.g., CD4+T, CD8+T, and B cells) and the secretion of immunomodulatory factors [13,14]. A randomized controlled trial (RCT) among pregnant women identified significant differences in DNA methylation patterns at delivery and 4–6 weeks postpartum between those supplemented with Vitamin D and the control group. The differentially methylated sites were predominantly involved in immune regulation pathways, suggesting that Vitamin D may influence immune function by regulating DNA methylation [15].

Building on emerging evidence that DNA methylation is a key regulator of immune function, this study investigates whether Vitamin D can mitigate PM-induced immune dysfunction in HIV-infected individuals, with a focus on DNA methylation as a key regulatory mechanism. The findings may offer novel insights into potential therapeutic strategies for enhancing immune resilience in this vulnerable population.

## 2. Materials and Methods

### 2.1. Study Design and Participants

We conducted a randomized, double-blind, placebo-controlled study across four administrative districts in Wuhan, China. The study protocol was registered with the Chinese Clinical Trial Registry (ChiCTR2300070301; registration date: 7 April 2023) and received approval from the Wuhan Center for Disease Control and Prevention (CDC) ethics committee (WHCDCIRB-K-2022027; approval date: 8 December 2022). After completing a baseline survey on 28 March 2023 and providing informed consent, 80 participants were randomly assigned at a 1:1 ratio to either the Vitamin D group (2 capsules daily, totaling 4000 IU, from Royal Oak Health Ltd., London, UK.) or the placebo group (2 capsules daily, containing custom-made soybean oil). The dose of 4000 IU/day was selected based on established safety guidelines and previous randomized controlled trials demonstrating its efficacy in increasing serum 25(OH)D levels with acceptable tolerability [16,17,18]. A total of four follow-up visits were conducted, with the final visit taking place on 18 July 2023. The randomization was conducted using a random number generator in R (version 4.2.0). The implementers of the program, data collectors at the four district-level CDCs, and the participants were blinded to the group assignments. Only the study’s project designer was aware of the allocation. Participants were selected according to the following inclusion/exclusion criteria.

The Inclusion Criteria: 1. HIV-infected individuals aged 18–65 years. 2. Stable on the same antiretroviral therapy regimen (tenofovir, lamivudine, and efavirenz) for at least one year. 3. No history of mental disorders, able to live independently, and no language barriers. 4. No chronic underlying diseases such as diabetes, hypertension, malignancies, thyroid disease, or coronary heart disease. Exclusion Criteria included: 1. Disease status progressed to the AIDS stage. 2. Left Wuhan within 15 days before follow-up. 3. Smokers (active smoking behavior within the past three months). 4. BMI > 28 kg/m^2^. 5. Regular alcohol consumption (at least once a week for the past three months or more than three times in the week before follow-up). 6. Suffering from autoimmune diseases such as systemic lupus erythematosus or rheumatoid arthritis. 7. Missed antiretroviral therapy medications for over seven days in the past 30 days. 8. Recently taking additional Vitamin D, beta-adrenergic blockers, statins, or other anti-inflammatory or antioxidant medications. 9. Currently experiencing active infections, inflammatory diseases, pregnancy, breastfeeding, severe diarrhea, or vomiting.

### 2.2. Procedures

The follow-up protocol is outlined in Figure S1. From the baseline survey to the fourth follow-up, participants took either Vitamin D or a placebo daily. The first follow-up took place 28 days after the baseline survey, with three additional follow-ups scheduled at four-week intervals. Atmospheric PM exposure was assessed starting on Thursday or Friday, with instruments operating for at least 72 h until the following Monday or Tuesday. During each follow-up, participants underwent physical examinations between 8:00 AM and 11:30 AM on either Monday or Tuesday, which included measurements of height and weight, questionnaire surveys, and fasting blood sample collections. Blood samples were collected using three EDTA anticoagulant tubes and one coagulation tube, with each tube collecting 5 mL of whole blood. This process was conducted by professional physicians from the district-level CDCs, who received standardized training and followed strict protocols to ensure data accuracy and reliability.

### 2.3. Outcomes

Our primary outcome was the lymphocyte subset counts, measured from blood samples collected at the end of weeks 4, 8, 12, and 16 from the start of the study. Anticoagulated whole blood was analyzed with a flow cytometer to determine CD3+T, CD4+T, CD8+T, CD19+B, natural killer (NK), and lymphocyte (LY) cell counts, utilizing the Agilent CD3/CD8/CD45/CD4 and Agilent CD3/CD16+CD56/CD45/CD19 Assay Kits. In addition, we measured plasma levels of immunomodulatory factors, including Toll-like receptor (TLR)-4, intercellular adhesion molecule (ICAM)-1, IL-6, tumor necrosis factor alpha (TNF-α), and interferon alpha-2 (IFNα2), using ELISA kits (Jianglai, Shanghai, China). Genomic DNA from whole blood was extracted using the DNeasy Blood & Tissue Kit (250, QIAGEN, Hilden, Germany), denatured using the EZ DNA Methylation-Gold Kit, and DNA methylation was assessed using the Illumina Infinium Methylation EPIC BeadChip. All tests followed the manufacturers’ instructions. Due to budget constraints, immunomodulatory factors and whole-genome DNA methylation were only measured in blood samples collected during follow-ups with the highest and lowest PM exposure concentrations for both groups. Serum 25(OH)D levels were measured at baseline and each follow-up to assess the effectiveness of Vitamin D supplementation and verify adherence in the intervention group while ensuring no additional Vitamin D supplementation in the placebo group. The levels of Serum 25(OH)D were quantified using an electrochemiluminescence immunoassay (Roche Diagnostics, Mannheim, Germany). Flow cytometry and serum 25(OH)D measurements were completed within 12 h of blood collection. Plasma samples for ELISA and whole blood samples for DNA methylation analysis were stored at −80 °C until subsequent analyses.

### 2.4. Exposure Measurements

**Figure 1:**
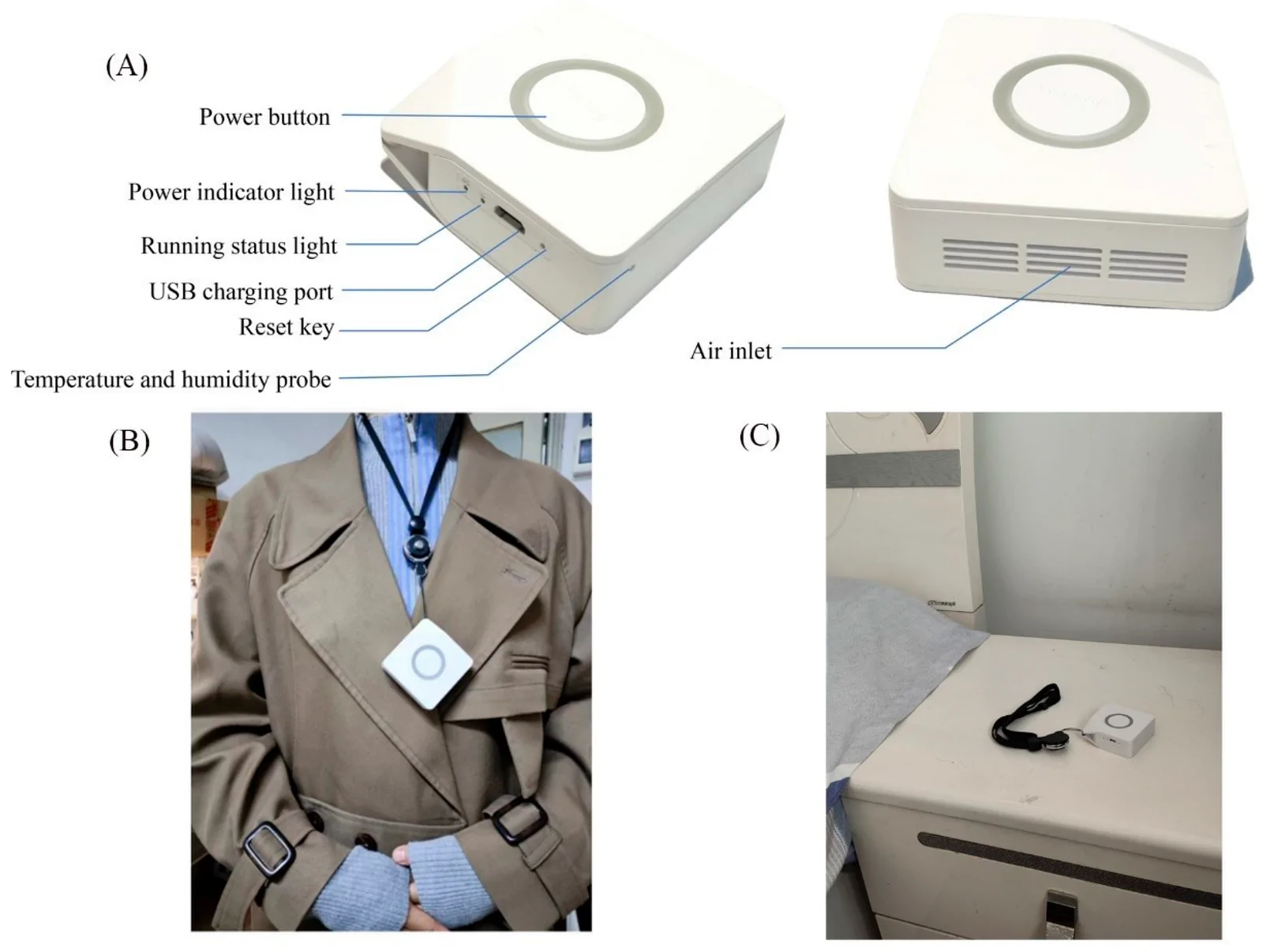
Optical detector for atmospheric particulate matter. Note: (**A**) Structure of the personal PM monitor. (**B**) Daytime wearing method. (**C**) Nighttime placement position.

Our team developed an atmospheric PM optical detection instrument called “Air Now” (Figure 1A). The device comprises a laser particle sensor (PMS7003, Plantower, Beijing, China) for fine PM detection and a temperature and humidity sensor (SHT30, Sensirion AG, Stäfa, Switzerland). The “Air Now” operates hourly, with each cycle lasting 30 s: the first 10 s clear residual air, and the remaining 20 s are used for real-time sampling and PM detection. Each operation produces seven data sets, including PM_1_, PM_2.5_, PM_10_, temperature, and relative humidity. Hourly data are obtained by averaging these seven sets. The portable PM monitor was compared with a DustTrak II Aerosol Monitor (Model 8532; TSI Inc., Shoreview, MN, USA), yielding a coefficient of determination of 0.993. The “Air Now” was used to measure environmental exposure conditions within 72 h before blood collection. Participants wore the instrument on their chests during the day (Figure 1B) and placed it bedside at night (Figure 1C) to ensure continuous hourly exposure data collection. Participants were instructed to maintain their usual daily activities without travel restrictions during the monitoring period to capture real-world personal PM exposure.

### 2.5. Covariates

Covariate data were mainly obtained from questionnaire surveys conducted at each follow-up. To ensure accuracy and consistency, all researchers involved in the surveys received standardized training to thoroughly understand the questionnaire content and master effective questioning techniques. Covariables in this study include age (years), sex, BMI, work types (outdoor worker, indoor worker, and mixed work), family income (<¥50,000, ¥50,000–100,000, ¥100,000–150,000, and >¥150,000), education level (junior, senior, and college or above), marital status (unmarried/divorced, and married), physical activity (low, moderate, and high), dietary habits (plant-based, balanced, and meat-based), frequency of drinking (no alcohol, 1–2 times per week, 3–4 times per week), emotional disorder (no, yes), sleep disorder (no, yes), temperature, and relative humidity. Physical activity was assessed using the Metabolic Equivalent of Task (MET) via the short form of the International Physical Activity Questionnaire (IPAQ), classifying participants into low, moderate, or high activity levels based on weekly activity. Sleep disorders were identified using the Athens Insomnia Scale (AIS), with scores exceeding 6 indicating a sleep disorder. Emotional disorders were evaluated using the Hospital Anxiety and Depression Scale, with scores above 11 for either HADS-A or HADS-D indicating emotional disorders.

### 2.6. Statistical Analysis

Sample size calculation formula is provided in the Supplementary Information Text S1. Continuous variables are presented as mean ± standard deviation, while categorical variables are reported as frequencies (percentages). Immune cell counts and immunomodulatory factors level were natural log-transformed to approximate a normal distribution, satisfying the linear model assumptions. Linear mixed-effects (LME) models were used to evaluate the association between PM exposure and immune cell counts in both groups. LME models were fitted using all available follow-up observations, allowing participants with incomplete repeated measurements to contribute to the analysis. Covariates adjusted in the models included age, sex, BMI, work type, family income, education level, marital status, physical activity, dietary habits, drinking frequency, emotional disorder, sleep disorder, temperature, and relative humidity. Temperature and relative humidity were incorporated into the models as natural spline functions with 3 degrees of freedom. Due to the short duration of the follow-up period, no time trend adjustments were applied. Variance Inflation Factors (VIFs) for all variables were assessed, with results indicating all VIFs were below 2, thus avoiding significant multicollinearity issues.

To explore the lag effect of PM exposure on immune cell counts, multiple exposure windows were analyzed, including 0–6 h, 7–12 h, 13–24 h, 25–48 h, 49–72 h, 0–12 h, 0–24 h, 0–48 h, and 0–72 h. Additionally, the study compared the estimated effect sizes of PM short-term exposure on immune cell counts between the two groups. The formulas used are as follows:

*d* = *β*_1_ − *β*_2_ (1)

(2) $$L=d-1.96\sqrt{{{\mathrm{se}}_{1}}^{2}+{{\mathrm{se}}_{2}}^{2}}$$

(3) $$U=d+1.96\sqrt{{{\mathrm{se}}_{1}}^{2}+{{\mathrm{se}}_{2}}^{2}}$$

where *β*_1_ and *β*_2_ represent the effect estimates of PM exposure for the Vitamin D group and placebo group, respectively. *se*_1_ and *se*_2_ represent the corresponding standard errors. *d* represents the average difference in effect estimates between the two groups, and *L* and *U* represent the lower and upper limits of the 95% CI of the mean difference between groups. For multiple comparisons of group differences, the Benjamini–Hochberg method was used to control the False Discovery Rate (FDR) across all tests involving six immune cell outcomes, three PM fractions, and nine exposure windows. An FDR-adjusted *p*-value < 0.05 was considered statistically significant. To assess the robustness of the findings, we additionally adjusted the LME models for baseline serum 25(OH)D concentrations, corresponding baseline immune cell counts, and both variables simultaneously. Furthermore, pooled linear mixed-effects models incorporating a PM exposure × vitamin D supplementation interaction term were fitted to evaluate between-group differences in PM-associated immune cell responses while accounting for repeated measurements.

For Genome-wide DNA methylation analysis, raw chip results underwent filtering, correction, and normalization. The filtering criteria included: (1) removal of probes with detected *p*-value ≥ 0.01 (6039 probes); (2) removal of probes with insufficient bead numbers (19,836 probes); (3) exclusion of non-CpG detection probes (2827 probes); (4) removal of multi-mapping probes (22 probes); (5) removal of probes with single nucleotide polymorphisms (95,116 probes); and (6) exclusion of probes on sex chromosomes X and Y (16,269 probes). The specific site cytosine methylation percentage (*β* value) is equivalent to the ratio of methylation (M) signal intensity to the sum of methylation (M) and non-methylation (U) signal intensities. The methylation *β*-value represents the proportion of DNA methylation (ranging from 0 to 1). Subsequently, *β* value matrix underwent normalization using Beta mixture quantile normalization to adjust for type I and type II probe biases [19]. Next, Singular Value Decomposition Analysis (SVA) was employed to examine batch effects arising from BeadChip Slide and Array, and Combat was applied for the subsequent correction of these batch effects [20]. A total of 64 samples and 726,017 probes were included in the subsequent analysis. M values, which were logit-transformed from the adjusted *β* values, were used as indicators of methylation levels. The transformation followed the formula:

(4) $$M=\log_{2}\left(\frac{\beta }{1-\beta }\right)$$

LME models were used to screen for differential CpG sites associated with Vitamin D intervention. The models were adjusted for sex, age, BMI, work type, education level, physical activity, frequency of drinking, atmospheric PM exposure, and directly measured leukocyte proportions composition (CD8+T, CD4+T, CD19+B, NK cells, monocytes, and granulocytes), with individuals included as random effects. An FDR-adjusted *p*-value < 0.05 was considered significant for identifying CpG sites significantly associated with Vitamin D intervention. Subsequently, LME models were used to analyze the association between the identified CpG sites and multiple immunomodulatory factors (including TLR4, ICAM-1, IL-6, TNF-α, and IFNα2), with significance defined as FDR-adjusted *p*-value < 0.05. Finally, mediation analysis explored the role of differential CpG sites in mediating the relationship between Vitamin D intervention and immunomodulatory factors, utilizing the “Mediation” package with 1000 bootstrap iterations to ensure robustness. Mediation proportions were calculated to assess the mediating effects. Additionally, enrichment analysis was performed on genes associated with differential CpG sites using clusterProfiler to identify relevant biological pathways, with significance set at *p* < 0.05. All statistical analyses were conducted using R software version 4.3.1, with the “minfi”, “IlluminaHumanMethylationEPICanno.ilm10b4.hg19”, “ChAMP”, “sva”, “variancePartition”, “lme4”, “mediation”, and “clusterProfiler” packages used for DNA methylation data processing and analysis.

## 3. Results

### 3.1. Participant Recruitment and Follow-Up

From 1 March 2023, to 18 July 2023, 80 HIV-infected individuals were recruited from four administrative districts in Wuhan, China. Five participants had only baseline data and did not complete any follow-up visits, including one who withdrew consent in the vitamin D group, three who relocated, and one who withdrew consent in the placebo group. Consequently, 75 participants (vitamin D: 39; placebo: 36) contributed a total of 271 follow-up observations to the longitudinal analysis. Throughout the study, 74 participants (Vitamin D: 38, placebo: 36) completed the first follow-up, 65 participants (Vitamin D: 35, placebo: 30) completed the second, 69 participants (Vitamin D: 35, placebo: 34) completed the third, and 63 participants (Vitamin D: 30, placebo: 33) completed the fourth follow-up (Figure S2).

### 3.2. Sample Baseline Characteristics

The basic characteristics of the participants were shown in Table 1. The average age of the 80 participants was 39.12 ± 15.69 years, with an average BMI of 22.32 ± 2.90 kg/m^2^. The study population was predominantly male (87.5%), with 61.3% indoor workers, 73.8% unmarried or divorced, and 60.0% having a college education or higher. Most participants maintained moderate levels of physical activity (51.3%) and balanced dietary habits (82.5%), with 72.5% never consuming alcohol. There were no statistically significant differences between the two groups regarding age, sex, BMI, work type, household income, education level, marital status, physical activity levels, dietary habits, drinking frequency, PSQI scores, HADS-A scores, HADS-D scores, serum 25(OH)D levels, and various immune cell counts.

### 3.3. Comparative Analysis of PM Exposure and Immunological Indicators Across Groups

**Figure 2:**
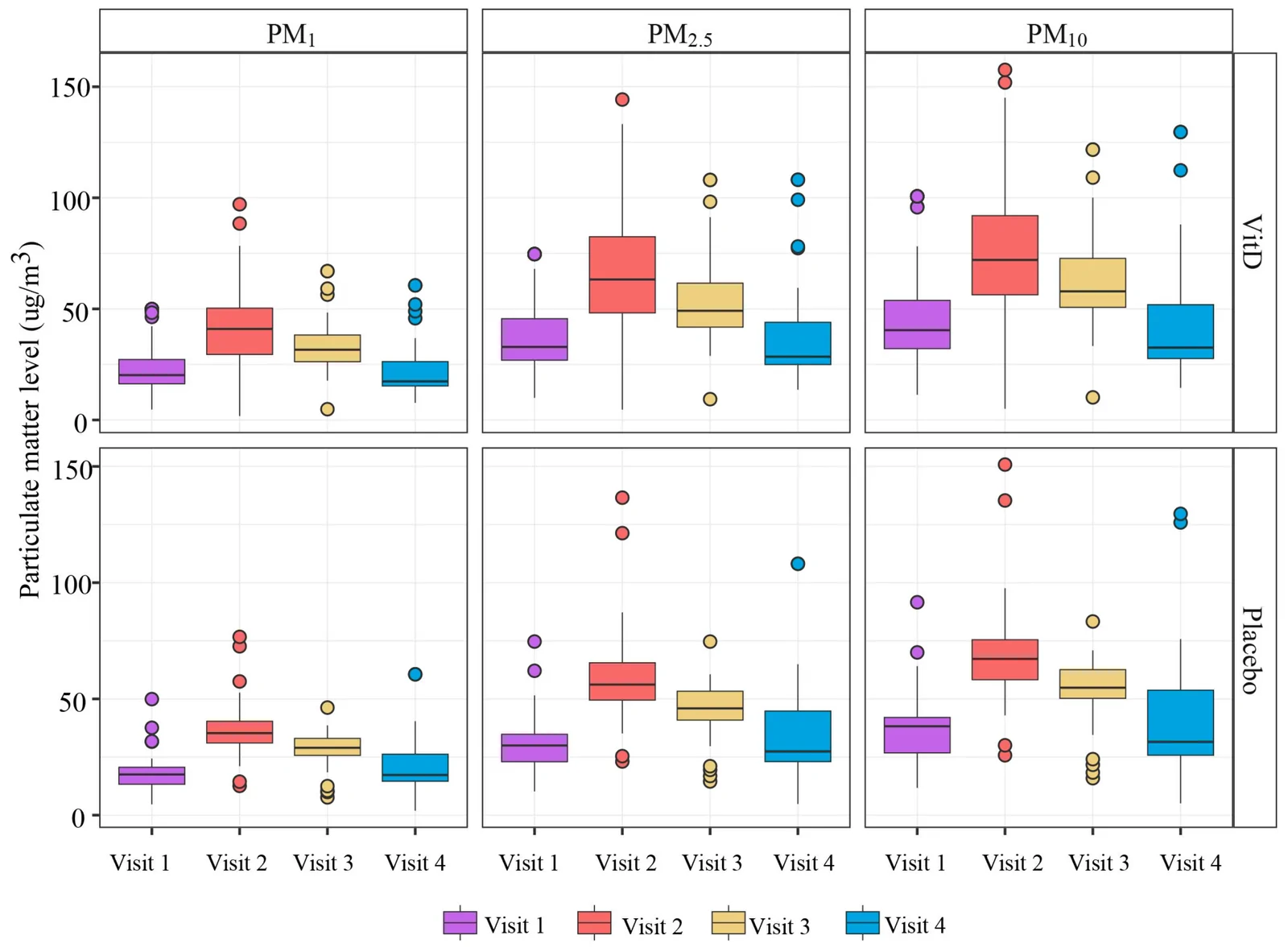
Mean 72-h exposure distribution prior to follow-up for both groups of participants.

PM concentrations varied across the four follow-up visits, with the highest concentrations observed at the second visit, followed by the third, fourth, and first visits (Figure 2). The average PM_2.5_ exposure concentrations for the 72 h before the first, second, third, and fourth follow-ups were 37.6 ± 26.8 µg/m^3^, 68.9 ± 57.7 µg/m^3^, 53.8 ± 38.7 µg/m^3^, and 39.8 ± 35.3 µg/m^3^, respectively. As shown in Figure S3, baseline serum 25(OH)D levels were 16.86 ± 5.50 ng/mL in the Vitamin D group and 19.52 ± 7.37 ng/mL in the placebo group. Serum 25(OH)D levels were significantly higher in the Vitamin D group than in the placebo group at all four follow-ups (*p* < 0.05). However, there were no significant differences in the counts of CD3+T, CD4+T, CD8+T, CD19+B, NK and LY between the two groups at the any follow-ups (Table S1). Table S2 demonstrates significant differences in TLR4 and IL-6 concentrations between the two groups at the visits during the lowest levels of PM exposure, as well as in TLR4 concentrations at visits during the highest levels of PM exposure (*p* < 0.05).

### 3.4. Comparative Effects of PM Exposure on Immune Cell Counts Between Vitamin D and Placebo Groups in HIV-Infected Individuals

**Figure 3:**
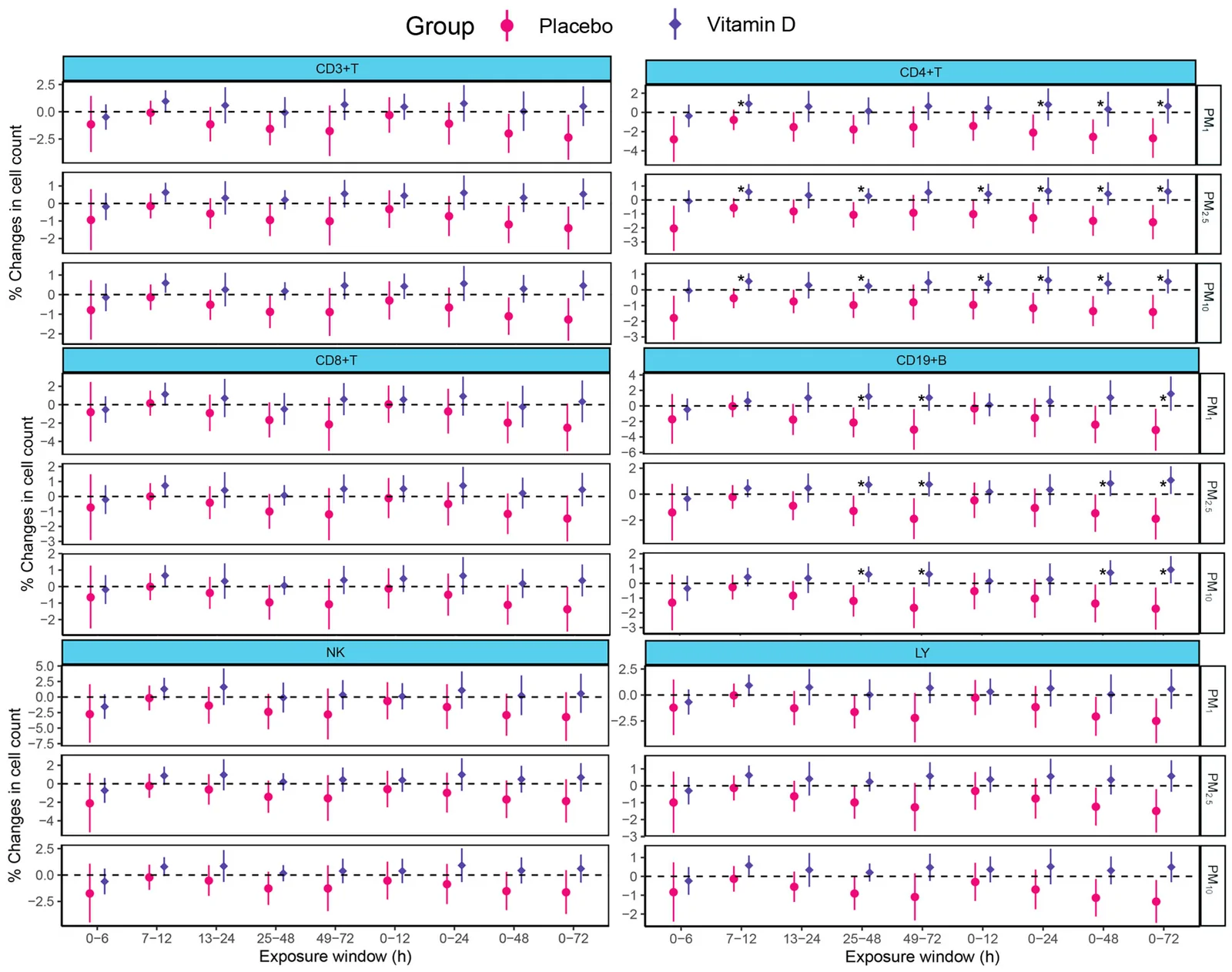
Associations between particulate matter concentrations measured during the preceding 72 h and immune cell counts in the vitamin D and placebo groups. Note: CD3+T, total T lymphocytes; CD4+T, helper T lymphocytes; CD8+T, suppressor T lymphocytes; CD19+B, total B lymphocytes; NK, natural killer cells; LY, lymphocytes. Models were adjusted for age, sex, BMI, work type, family income, education level, marital status, physical activity, dietary habits, drinking frequency, emotional disorder, sleep disorder, temperature, and relative humidity. The horizontal dashed line at zero corresponds to the null effect. * indicates a statistically significant between-group difference in PM-associated regression coefficients (BH-FDR-adjusted *p* < 0.05).

As shown in Figure 3, in the placebo group, PM exposure was negatively correlated with percentage changes in CD3+T (25–48 h, 0–48 h, 0–72 h), CD4+T (0–6 h, 25–48 h, 0–24 h, 0–48 h, 0–72 h), CD19+B (25–48 h, 49–72 h, 0–48 h, 0–72 h), and LY (25–48 h, 0–48 h, 0–72 h) cell counts. During the 0–72 h exposure window, each 10 µg/m^3^ increase in PM_1_, PM_2.5_, and PM_10_ concentrations was associated with decreases in CD3+T cell counts by 2.36%, 1.41%, and 1.27%; CD4+T cell counts by 2.69%, 1.59%, and 1.41%; CD19+B cell counts by 3.11%, 1.90%, and 1.72%; and LY cell counts by 2.50%, 1.49%, and 1.34%, respectively. Furthermore, significant differences between the two groups were found in the effects of atmospheric PM exposure on CD4+T cell counts (7–12 h, 0–12 h, 25–48 h, 0–24 h, 0–48 h, 0–72 h) and CD19+B cell counts (25–48 h, 49–72 h, 0–48 h, 0–72 h) (FDR-adjusted *p* < 0.05). Specifically, compared with the placebo group, the estimated PM-associated changes in CD4+T cell counts in the vitamin D group differed by 3.37% (95% CI: 0.58%, 6.16%), 2.21% (95% CI: 0.68%, 3.74%), and 1.96% (95% CI: 0.61%, 3.30%) per 10 µg/m^3^ increase in PM_1_, PM_2.5_, and PM_10_ concentrations, respectively. The corresponding differences for CD19+B cell counts were 4.71% (95% CI: 1.17%, 8.26%), 2.98% (95% CI: 1.03%, 4.94%), and 2.65% (95% CI: 0.94%, 4.37%), respectively.

Additional adjustment for baseline 25(OH)D concentrations, corresponding baseline immune cell counts, or both yielded between-group differences in PM-associated CD4+T and CD19+B cell responses that were broadly consistent in direction and magnitude with the primary analysis. For the 0–72 h exposure window, these differences remained significant after within-outcome BH-FDR correction in all three models (Tables S3–S5). In pooled linear mixed-effects models, nominally significant PM × treatment interactions were observed for CD4+T and CD19+B cell counts with PM_2.5_ and PM_10_ during the 0–48 h and 0–72 h exposure windows, as well as for CD19+B cell counts with PM_1_ during 0–72 h (all unadjusted *p* < 0.05). However, none of these interactions remained statistically significant after BH-FDR correction (Table S6).

### 3.5. Association of Differentially Methylated Sites with Immunomodulatory Factors

**Figure 4:**
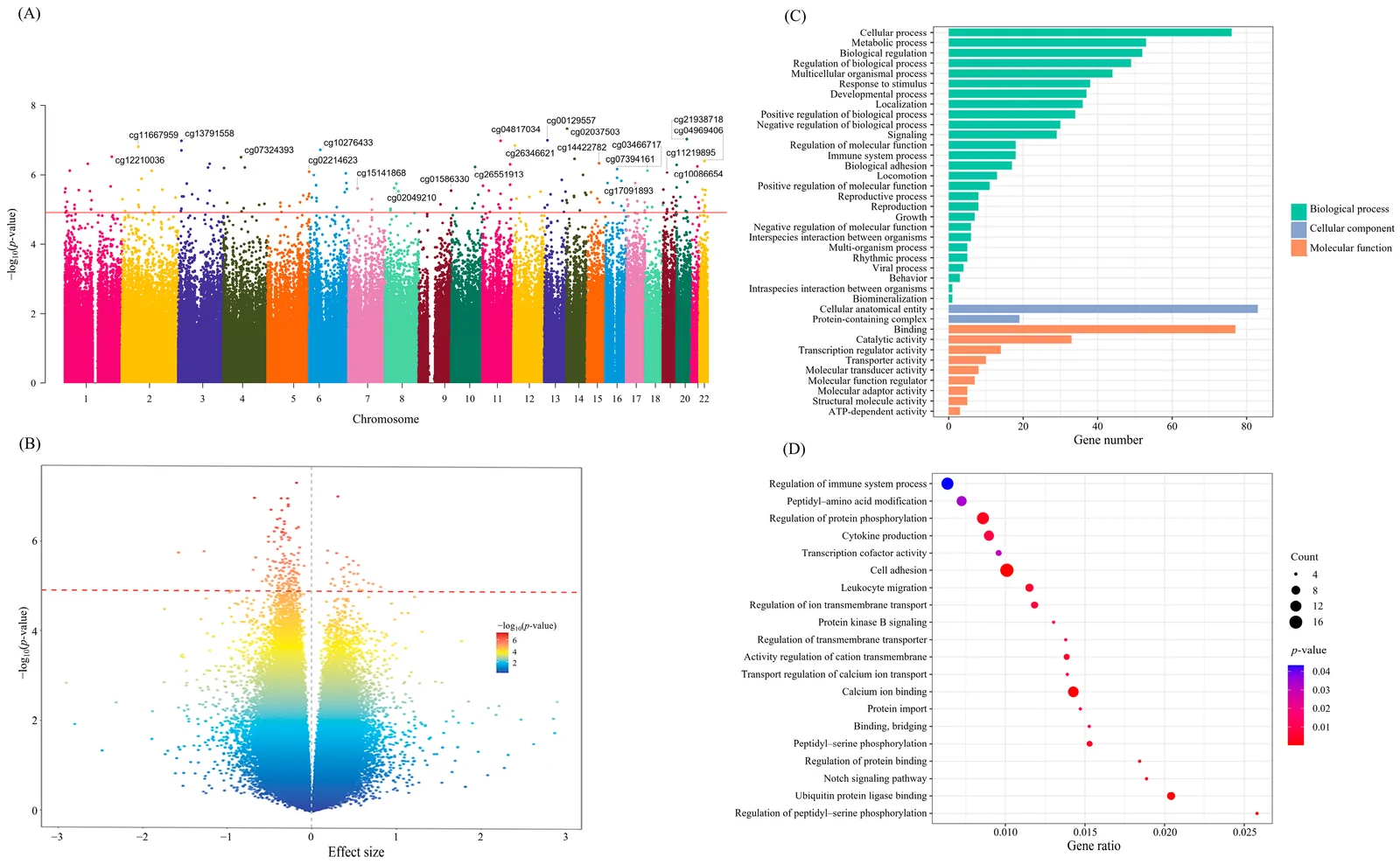
Identification and enrichment analysis of differentially methylated sites associated with Vitamin D intervention. Note: (**A**) Manhattan plot of differentially methylated CpG sites associated with Vitamin D intervention. The red dashed line denotes the genome-wide significance threshold. (**B**) Volcano plot depicting the distribution of differentially methylated CpG sites associated with Vitamin D intervention. The red dashed line marks the genome-wide significance threshold. (**C**) Annotated gene GO analysis of differentially methylated CpG sites. (**D**) GO pathway bubble map of annotated genes with differentially methylated CpG sites.

Based on the initial 726,017 CpG sites, a Manhattan plot was generated (Figure 4A); this identified 173 significantly differentially methylated sites between the Vitamin D group and the placebo group (Table S7). A volcano plot highlighted 146 hypomethylated and 27 hypermethylated sites in the Vitamin D group compared to the placebo group (Figure 4B). The top five CpG sites ranked by *p*-value were cg02037503, cg21938718, cg00129557, cg13791558, and cg04817034. The methylation M-values of 52 CpG sites were positively correlated with TLR4 concentrations, with regression coefficients (β) of 1.55 (95% CI: 1.01, 2.09) for cg23096476, 0.97 (95% CI: 0.56, 1.37) for cg04982488, and 0.67 (95% CI: 0.36, 0.99) for cg27066321. Conversely, methylation levels of 10 CpG sites were negatively correlated with TLR4 concentrations, including cg08558340 (β = −1.14, 95% CI: −1.58, −0.69), cg00830604 (β = −0.68, 95% CI: −1.11, −0.24), and cg26014511 (β = −0.54, 95% CI: −0.99, −0.09) (Table S8). Additionally, the study also identified 3 CpG sites associated with ICAM-1 concentrations, 56 with IL-6, 96 with TNF-α, and 15 with IFNα2. (Tables S9–S12). GO enrichment analysis of the 93 genes annotated by the 137 differentially methylated CpG sites indicated significant involvement in biological pathways related to immune regulation, including cell adhesion, Notch signaling pathway, leukocyte migration, cytokine production, ubiquitin protein ligase binding, regulation of protein phosphorylation, and regulation of immune system processes (Figure 4C,D).

### 3.6. Mediation of DNA Methylation in the Association Between Vitamin D Intervention and Immunomodulatory Factors

Exploratory mediation analyses identified five CpG sites showing statistically significant estimated indirect effects in the association between vitamin D supplementation and TLR4 concentrations (Table 2). The mediation effects of cg27066321 (β: −0.278, 95% CI: −0.495, −0.091), cg04982488 (β: −0.338, 95% CI: −0.588, −0.145), cg23096476 (β: −0.584, 95% CI: −1.027, −0.214), cg25499646 (β: −0.268, 95% CI: −0.533, −0.021), and cg08558340 (β: −0.475, 95% CI: −0.887, −0.138) accounted for 36.97%, 42.44%, 77.06%, 33.79%, and 63.82% of the total effect, respectively. The methylation levels of 10 CpG sites, including cg08500112, cg17164356, cg05990080, cg14070323, cg23096476, cg11304950, cg11962082, cg09996840, cg25317338, and cg05015022, mediated the reduction in IL-6 levels due to Vitamin D intervention by contributing 34.63%, 37.12%, 34.74%, 27.98%, 78.78%, 36.26%, 62.83%, 47.08%, 50.81%, and 36.92% of the mediation effect, respectively. Furthermore, 12 CpG sites, including cg25882580, cg06709331, and cg23528791, etc., mediated the association between Vitamin D intervention and TNF-α levels, with mediation proportions ranging from 57.09% to 76.86%.

## 4. Discussion

This study identified negative associations between PM concentrations measured during recent exposure windows and CD3+T cell, CD4+T cell, CD19+B cell, and lymphocyte counts. Vitamin D supplementation was associated with attenuated negative PM-related associations for CD4+T cell and CD19+B cell counts.

Previous studies show that PM exposure alters immune cell counts in vulnerable populations. For instance, a cohort of 1397 infants found that a 25 µg/m^3^ increase in PM_2.5_ before birth reduced CD4+T cells by 2.27% and increased CD19+B cells by 1.55%, without affecting other cell types [21]. Another study indicated that every 10 µg/m^3^ increase in PM_2.5_ and PM_10_ exposure during early pregnancy decreased CD3+T and CD8+T cell proportions by 2.69% and 1.84% (PM_2.5_), and by 1.72% and 1.37% (PM_10_), respectively, in 3-year-old children, with no association observed for CD4+T cell [22]. Our findings align with these results, showing a negative association between PM exposure and CD3+T, CD4+T, CD19+B, and LY cell counts, but no effect on NK cells. These findings suggest that PM exposure may adversely affect immune function by selectively influencing immune cell populations in vulnerable individuals. When interpreting these associations, the observed PM exposure levels should also be considered. Mean 72-h PM_2.5_ concentrations ranged from 37.6 to 68.9 µg/m^3^ across follow-up visits, indicating variability in personal exposure during the study period. As PM exposure was measured continuously without predefined high- or low-exposure groups, these findings should be interpreted within the observed exposure range.

Vitamin D supplementation appeared to attenuate the negative associations between PM exposure and CD4+T cell and CD19+B cell counts in individuals living with HIV. This aligns with findings in healthy populations, where Vitamin D modulates immune cell function and inflammation [23]. Unlike transient immune responses in healthy individuals, HIV-infected individuals may benefit more from Vitamin D’s ability to enhance immune resilience and reduce inflammation. Vitamin D exerts protective effects against PM-induced immune dysfunction through its anti-inflammatory and immunomodulatory properties [9]. The active metabolite, 1,25(OH)_2_D_3_, suppresses NF-κB signaling in lymphocytes, thereby reducing pro-inflammatory cytokine production and limiting immune dysregulation [24,25,26]. This action helps to counteract the immune dysregulation and cellular damage that often follow PM exposure. Additionally, Vitamin D decreases B cell antigen presentation, dampening T-cell inflammatory responses and promoting immune stability [27]. In HIV-infected individuals, Vitamin D enhances immune defense by inducing antiviral gene expression, downregulating CCR5 on CD4+T cells, and preserving T cell integrity [28]. Through VDR-mediated pathways, it further promotes monocyte-to-macrophage differentiation and enhances antimicrobial responses, underscoring its role in maintaining immune homeostasis under environmental stress [29,30]. These mechanisms may help explain the observed differences in PM-associated immune cell responses between the intervention groups.

The potential influence of antiretroviral therapy (ART) should also be considered when interpreting these findings. Efavirenz-containing regimens have been associated with reduced circulating 25(OH)D concentrations, potentially through altered vitamin D metabolism [31]. In our study, all participants had received the same tenofovir, lamivudine, and efavirenz regimen for at least one year before enrollment, minimizing heterogeneity in ART exposure between groups. Notably, serum 25(OH)D concentrations were consistently higher in the vitamin D group throughout follow-up, indicating a biochemical response to supplementation within this treatment context. This finding is consistent with a previous randomized trial showing that efavirenz use did not diminish the 25(OH)D response to vitamin D supplementation [32]. Nevertheless, the potential influence of ART on immune responses and the generalizability of our findings to other ART regimens remain uncertain. Although our findings suggest that vitamin D supplementation may attenuate PM-associated immune alterations, their direct clinical implications remain uncertain. Nevertheless, these observations provide preliminary evidence to inform future investigations into strategies for protecting immune health in people living with HIV exposed to air pollution. Further studies incorporating clinical outcomes are needed to determine whether these associations translate into meaningful health benefits.

We identified 173 differentially methylated CpG sites linked to Vitamin D supplementation, with 84.39% (146 sites) showing hypomethylation, annotated to 122 genes. These findings suggest that vitamin D supplementation may be associated with changes in the blood DNA methylation landscape. Notably, GO enrichment analysis identified biological processes related to leukocyte migration, cell adhesion, cytokine production, Notch signaling, and immune regulation. These processes are relevant to immune cell trafficking, inflammatory signaling, and immune homeostasis, providing a biological context for the observed methylation differences. Our findings align with Cruz et al., who reported methylation changes at specific CpG sites (cg00523161, cg21165262, cg07145979, and cg13918754) in genes like *LPIN1*, *DYSF*, *COL23A1*, and *RNF39* among individuals taking Vitamin D [33], whereas another randomized trial reported differential methylation at a CpG site annotated to *GAB2* [15]. LPIN1 has been implicated in metabolic and inflammatory regulation [34,35,36], while GAB2 participates in intracellular signaling pathways associated with immune responses [37,38]. These findings support the biological plausibility of an association between vitamin D supplementation and epigenetic variation in immune-related processes. 

At the candidate-gene level, exploratory mediation analyses identified several CpG sites showing potential indirect effects in associations between vitamin D supplementation and immunomodulatory factors. Among these, cg27066321 was annotated to *PEX14*, a gene involved in peroxisomal protein import and homeostasis. Previous studies have demonstrated the importance of peroxisomal regulation in macrophage redox balance and inflammatory responses [39]. Notably, experimental induction of peroxisomes attenuated lipopolysaccharide -induced production of COX-2 and pro-inflammatory cytokines, including TNF-α, IL-6, and IL-12, whereas *PEX14* knockdown enhanced lipopolysaccharide induced inflammatory responses [40]. These findings provide a biological rationale for investigating the potential involvement of PEX14 in TLR4-related immune regulation. Another candidate, cg04982488, was annotated to *AFAP1L2*, which encodes the adaptor protein XB130. XB130 participates in Src-related intracellular signaling [41], and experimental deletion of XB130 has been associated with enhanced inflammatory responses, including increased NF-κB activity and ERK1/2 phosphorylation in a skin inflammation model [42]. Separately, the p38γ/δ–ERK1/2 signaling axis has been shown to regulate TLR4-induced cytokine production [43], although a direct mechanistic connection between AFAP1L2 and this pathway remains unestablished. In addition, cg08500112, annotated to *PDE4D*, was identified in the exploratory analysis involving IL-6. The PDE4 family regulates intracellular cAMP signaling, which plays an important role in inflammatory cytokine production [44]. Together, these findings suggest several biologically plausible pathways that may be relevant to the observed epigenetic associations. However, the functional consequences of the identified CpG sites remain uncertain, as gene expression and protein activity were not directly assessed. Further integrated methylation–transcriptomic and experimental studies are needed to validate these candidate mechanisms.

This study offers several strengths, including the use of individual-level PM exposure monitoring to precisely assess its impact on immune function, and the first RCT to investigate genome-wide DNA methylation changes induced by Vitamin D supplementation in HIV-infected individuals. The exploratory mediation analyses identified candidate CpG sites potentially associated with indirect immunomodulatory effects, providing hypotheses for future mechanistic investigations. However, several limitations should be emphasized when interpreting the results. First, the relatively small sample size may limit the accuracy and power of the trial. Increasing the sample size in future studies could enhance the robustness of the findings. Second, the male-to-female ratio of HIV-infected individuals in Wuhan is approximately 10:1, and this study could not recruit participants at a 1:1 gender ratio. However, the gender ratio difference between the two groups was not statistically significant. Future research should aim for a more balanced gender ratio. Third, this study did not measure the expression of genes annotated by differentially methylated CpG sites identified through DNA methylation chips. Future studies could functionally validate these annotated genes. Fourth, although the mediation analyses identified potential indirect effects of DNA methylation, these findings should be interpreted cautiously. DNA methylation was not randomized, and its concurrent measurement with immunomodulatory factors prevented confirmation of temporal precedence. Residual confounding and alternative biological mechanisms cannot be excluded, and causal mediation cannot be established. Finally, PM exposure was assessed within 72 h before each follow-up visit, which captured recent exposure levels but did not account for participants’ long-term exposure histories. Therefore, the observed associations cannot be attributed exclusively to recent PM exposure, as potential contributions from cumulative or long-term exposure cannot be excluded.

## 5. Conclusions

In conclusion, vitamin D supplementation was associated with attenuated negative associations between recently measured PM concentrations and CD4+T cell and CD19+B cell counts in people living with HIV. Exploratory DNA methylation analyses identified potential epigenetic associations with immunomodulatory responses. These findings provide novel insights into the epigenetic pathways underlying immune dysfunction and offer implications for targeted interventions in vulnerable populations.

## Figures and Tables

**Table 1:** Baseline characteristics of the subjects.

Characteristic	Total	Vitamin D Group	Placebo Group	*p*-Value
	*N* = 80	*N* = 40	*N* = 40	
Sex, *n* (%)				0.735
Male	70 (87.5)	36 (90.0)	34 (85.0)	
Female	10 (12.5)	4 (10.0)	6 (15.0)	
Age, years, Mean (SD)	39.12 (15.69)	38.73 (16.06)	39.53 (15.51)	0.821
BMI, kg/m^2^, Mean (SD)	22.32 (2.90)	21.96 (3.19)	22.69 (2.56)	0.265
Work types, *n* (%)				0.786
Outdoor worker	7 (8.75)	3 (7.50)	4 (10.0)	
Indoor worker	49 (61.3)	26 (65.0)	23 (57.5)	
Mixed work	24 (30.0)	11 (27.5)	13 (32.5)	
Family income, *n* (%)				0.584
<¥50,000	33 (41.2)	19 (47.5)	14 (35.0)	
¥50,000–100,000	26 (32.5)	11 (27.5)	15 (37.5)	
¥100,000–150,000	15 (18.8)	8 (20.0)	7 (17.5)	
>¥150,000	6 (7.50)	2 (5.00)	4 (10.0)	
Education level, *n* (%)				0.513
Junior	16 (20.0)	8 (20.0)	8 (20.0)	
Senior	16 (20.0)	10 (25.0)	6 (15.0)	
College or above	48 (60.0)	22 (55.0)	26 (65.0)	
Marital status, *n* (%)				0.611
Unmarried/widowed	59 (73.8)	28 (70.0)	31 (77.5)	
Married	21 (26.2)	12 (30.0)	9 (22.5)	
Physical activity, *n* (%)				0.498
Low	17 (21.2)	9 (22.5)	8 (20.0)	
Moderate	41 (51.3)	18 (45.0)	23 (57.5)	
High	22 (27.5)	13 (32.5)	9 (22.5)	
Dietary habits, *n* (%)				0.457
Plant-based diet	9 (11.2)	4 (10.0)	5 (12.5)	
Balanced diet	66 (82.5)	32 (80.0)	34 (85.0)	
Meat-based diet	5 (6.25)	4 (10.0)	1 (2.50)	
Frequency of drinking, *n* (%)				0.124
No alcohol	58 (72.5)	28 (70.0)	30 (75.0)	
1–2 times	19 (23.8)	12 (30.0)	7 (17.5)	
3–4 times	3 (3.75)	0 (0.00)	3 (7.50)	
PSQI score	6.30 (3.68)	5.90 (2.99)	6.70 (4.26)	0.334
HADS-A score, Mean (SD)	5.42 (3.47)	5.22 (3.19)	5.62 (3.76)	0.609
HADS-D score, Mean (SD)	4.94 (2.87)	4.62 (3.07)	5.25 (2.67)	0.334
CD3+T, cell/ul, Mean (SD)	1363 (495)	1291 (491)	1434 (495)	0.199
CD4+T, cell/ul, Mean (SD)	532 (217)	516 (226)	548 (210)	0.520
CD8+T, cell/ul, Mean (SD)	717 (343)	660 (329)	773 (352)	0.144
CD19+B, cell/ul, Mean (SD)	197 (107)	197 (116)	197 (99.0)	0.992
NK, cell/ul, Mean (SD)	373 (223)	327 (191)	419 (245)	0.066
LY, cell/ul, Mean (SD)	1968 (661)	1845 (633)	2092 (672)	0.096

**Table 2:** The mediating effect of DNA methylation in the association between Vitamin D intervention and immunomodulatory factors.

Immunomodulatory Factors	CpG	Gene	Direct Effect Estimate (95% CI)	Indirect Effect Estimate (95% CI)	Proportion Mediated (%)	*p*-Mediation
TLR4	cg27066321	PEX14	−0.470 (−0.856, −0.097)	−0.278 (−0.495, −0.091)	36.97	<0.001
	cg04982488	AFAP1L2	−0.449 (−0.794, −0.074)	−0.338 (−0.588, −0.145)	42.44	<0.001
	cg23096476	CBS	−0.170 (−0.674, 0.309)	−0.584 (−1.027, −0.214)	77.06	<0.001
	cg25499646	ATP8B3	−0.514 (−0.926, −0.095)	−0.268 (−0.533, −0.021)	33.79	0.034
	cg08558340	SRRT	−0.277 (−0.780, 0.208)	−0.475 (−0.887, −0.138)	63.82	0.004
IL-6	cg08500112	PDE4D	−0.317 (−0.589, −0.049)	−0.174 (−0.325, −0.050)	34.63	0.008
	cg17164356	TCP1	−0.294 (−0.579, −0.019)	−0.172 (−0.331, −0.042)	37.12	0.008
	cg05990080	CTDSPL	−0.320 (−0.620, −0.027)	−0.169 (−0.326, −0.028)	34.74	0.018
	cg14070323	MEG3	−0.368 (−0.660, −0.098)	−0.144 (−0.283, −0.017)	27.98	0.018
	cg23096476	CBS	−0.098 (−0.438, 0.249)	−0.383 (−0.691, −0.142)	78.78	0.002
	cg11304950	BST1	−0.272 (−0.578, 0.033)	−0.153 (−0.290, −0.046)	36.26	0.002
	cg11962082	FAM184A	−0.185 (−0.522, 0.125)	−0.317 (−0.581, −0.087)	62.83	0.004
	cg09996840	SLC25A10	−0.243 (−0.573, 0.092)	−0.231 (−0.459, −0.044)	47.08	0.020
	cg25317338	MIR7853; NCKAP5	−0.223 (−0.598, 0.129)	−0.242 (−0.499, −0.033)	50.81	0.028
	cg05015022	FRMD6	−0.303 (−0.654, 0.021)	−0.172 (−0.344, −0.016)	36.92	0.032
TNF-α	cg25882580	CACNG6	−0.083 (−0.363, 0.194)	−0.239 (−0.434, −0.089)	74.18	0.008
	cg06709331	CLDN15	−0.093 (−0.403, 0.200)	−0.219 (−0.399, −0.064)	68.44	0.022
	cg23528791	CNKSR3	−0.094 (−0.379, 0.185)	−0.226 (−0.413, −0.069)	69.55	0.022
	cg17141215	TMEM40	−0.120 (−0.400, 0.163)	−0.199 (−0.375, −0.049)	61.53	0.024
	cg14565651	SLC2A5	−0.131 (−0.411, 0.138)	−0.198 (−0.358, −0.063)	60.71	0.016
	cg10142237	CSNK1E	−0.119 (−0.430, 0.188)	−0.215 (−0.413, −0.053)	63.96	0.024
	cg27493649	RNF39	−0.103 (−0.417, 0.191)	−0.224 (−0.432, −0.059)	68.45	0.030
	cg25499646	ATP8B3	−0.080 (−0.421, 0.236)	−0.245 (−0.486, −0.056)	76.86	0.034
	cg07593390	IL20RB	−0.117 (−0.437, 0.188)	−0.208 (−0.413, −0.018)	64.49	0.040
	cg01868616	ARRB1	−0.125 (−0.431, 0.175)	−0.182 (−0.372, −0.020)	58.16	0.042
	cg10066188	NAT8B	−0.133 (−0.429, 0.169)	−0.182 (−0.347, −0.031)	57.09	0.042
	cg12210036	GOLT1A	−0.070 (−0.401, 0.267)	−0.254 (−0.450, −0.075)	75.90	0.046

Note: The mediation model refers to the linear mixed-effects model examining the as sociation between Vitamin D intervention and DNA methylation levels. The outcome model refers to the linear mixed-effects model assessing the associations between Vitamin D intervention, DNA methylation levels, and immunomodulatory factors concentrations. Both models were adjusted for sex, age, body mass index, work type, education level, physical activity, frequency of drinking, particulate matter exposure, and leukocyte composition.

## Data Availability

The datasets used and/or analyzed during the current study are available from the corresponding author on reasonable request.

## Supplementary Materials

The following supporting information can be downloaded at: https://www.mdpi.com/article/10.3390/nu18213710/s1, Supplementary Information Text S1. Figure S1: Follow-up protocol of the study. Figure S2: Participant flow through the trial. Figure S3: Distribution of serum 25(OH)D concentrations in the two study groups during the follow-up period. Table S1: Levels of lymphocyte counts during follow-up of study subjects. Table S2: Distribution of immunomodulatory factors in subjects under the low and high PM exposure visit. Table S3: PM-associated changes in CD4+T and CD19+B cell counts in the vitamin D and placebo groups with additional adjustment for baseline 25(OH)D. Table S4: PM-associated changes in CD4+T and CD19+B cell counts in the vitamin D and placebo groups with additional adjustment for the corresponding baseline immune cell count. Table S5: PM-associated changes in CD4+T and CD19+B cell counts in the vitamin D and placebo groups with additional adjustment for baseline 25(OH)D and the corresponding baseline immune cell count. Table S6: PM-associated changes in CD4+T and CD19+B cell counts and PM-by-treatment interactions. Table S7: Association between Vitamin D Intervention and Whole-Genome DNA Methylation in people living with HIV. Table S8: Association between Vitamin D intervention-related CpGs and TLR4 concentrations (Top 20 ranked by *p*-value). Table S9: Association between Vitamin D intervention-related CpGs and ICAM-1 Concentrations (Top 20 ranked by *p*-value). Table S10: Association between Vitamin D intervention-related CpGs and IL-6 Concentrations (Top 20 ranked by *p*-value). Table S11: Association between Vitamin D intervention-related CpGs and TNF-α concentrations (Top 20 ranked by *p*-value). Table S12: Association between Vitamin D intervention-related CpGs and IFNα2 concentrations (Top 20 ranked by *p*-value).

## Abbreviations

The following abbreviations are used in this manuscript:

PM	particulate matter
PM_1_	submicron particulate matter
PM_2.5_	fine particulate matter
PM_10_	inhalable particulate matter
HIV	human immunodeficiency virus
AIDS	acquired immunodeficiency syndrome
RCT	randomized controlled trial
CDC	Centers for Disease Control and Prevention
BMI	body mass index
SD	standard deviation
CI	confidence interval
LME	linear mixed-effects model
VIF	variance inflation factor
FDR	false discovery rate
SVA	singular value decomposition analysis
GO	Gene Ontology
25(OH)D	25-hydroxyvitamin D
CD3+T	total T lymphocytes
CD4+T	helper T lymphocytes
CD8+T	suppressor T lymphocytes
CD19+B	total B lymphocytes
NK	natural killer cells
LY	lymphocytes
TLR4	Toll-like receptor 4
ICAM-1	intercellular adhesion molecule-1
IL-6	interleukin-6
TNF-α	tumor necrosis factor-α
IFNα2	interferon alpha-2
MET	Metabolic Equivalent of Task
IPAQ	International Physical Activity Questionnaire
AIS	Athens Insomnia Scale
HADS	Hospital Anxiety and Depression Scale
PSQI	Pittsburgh Sleep Quality Index
