# Supplementary material for: Vitamin D and Particulate Matter–Associated Immune Responses in People Living with HIV: A Randomized Trial with Exploratory DNA Methylation Analyses
Source: Nutrients. 2026 Nov 9;18(21):3710. doi: 10.3390/nu18213710 (PMC13765612; doi:10.3390/nu18213710)
Supplement: Supplementary file 1 [file nutrients-18-03710-s001.zip › nutrients-4660205-supplementary.pdf]

Vitamin D and Particulate Matter–Associated Immune Responses in People Living With HIV: A Randomized Trial with Exploratory DNA Methylation Analyses

Huimin Ben[1†], Yuqian Hua[1†], Zixuan Ping[1], Shouxin Peng[2], Yuxuan Tan[3], Xiuli Zhao[1], ZhiHao Xie[1], Xiaoxia Liu[4*], Wei Liang[1*]

Contents

Supplementary Information Text S1

Figure S1 Follow-up protocol of the study.

Figure S2 Participant flow through the trial.

Figure S3 Distribution of serum 25(OH)D concentrations in the two study groups during the follow up period.

Table S1 Levels of lymphocyte counts during follow-up of study subjects.

Table S2 Distribution of immunomodulatory factors in subjects under the low and high PM exposure visit.

Table S3 PM-associated changes in CD4+T and CD19+B cell counts in the vitamin D and placebo groups with additional adjustment for baseline 25(OH)D.

Table S4 PM-associated changes in CD4+T and CD19+B cell counts in the vitamin D and placebo groups with additional adjustment for the corresponding baseline immune cell count.

Table S5 PM-associated changes in CD4+T and CD19+B cell counts in the vitamin D and placebo groups with additional adjustment for baseline 25(OH)D and the corresponding baseline immune cell count.

Table S6 PM-associated changes in CD4+T and CD19+B cell counts and PM-by-treatment interactions.

Table S7 Association between Vitamin D Intervention and Whole-Genome DNA Methylation in people living with HIV.

Table S8 Association between Vitamin D intervention-related CpGs and TLR4 concentrations (Top 20 ranked by *P*-value).

Table S9 Association between Vitamin D intervention-related CpGs and ICAM-1 Concentrations (Top 20 ranked by *P*-value).

Table S10 Association between Vitamin D intervention-related CpGs and IL-6 Concentrations (Top 20 ranked by *P*-value).

Table S11 Association between Vitamin D intervention-related CpGs and TNF-α concentrations (Top 20 ranked by *P*-value).

Table S12 Association between Vitamin D intervention-related CpGs and IFNα2 concentrations (Top 20 ranked by *P*-value).

Supplementary Information Text S1

Estimation of sample size

The sample size was estimated using the calculation formula of repeated measurement design studies validated and recommended in the Analysis of longitudinal data：

$$N = \frac{2(Z_\alpha + Z_\beta)^2[1+(n-1)\rho]}{n[(\mu_1 - \mu_2)/\sigma]^2} \qquad \text{Equation（S1）}$$

N is required sample size，Z_α and Z_β represent the critical values of the normal distribution，σ is the inter-group standard deviation，n is the number of repeated measurements，and ρ is the intraclass correlation coefficient. $(\mu_1 - \mu_2)/\sigma$ represents the smallest detectable difference between groups. Give $\alpha = 0.05$, $1\text{-}\beta = 0.8$ four repeated measurements, and referencing a study on the association between Vitamin D intervention and CD4+ T cell counts in AIDS patients [S1], where $(\mu_1 - \mu_2)/\sigma$ is calculated as 0.52 and ρ as 0.4，the required minimum sample size is 32. Accounting for a 20% dropout rate, each group required at least 38 participants. Therefore, 40 participants were recruited per group, resulting in a total of 80 HIV/AIDS patients for the study.

Reference

[S1] Falasca K, Ucciferri C, Di Nicola M, et al. Different strategies of 25OH vitamin D supplementation in HIV-positive subjects. Int J STD AIDS, 2014, 25(11): 785-792.

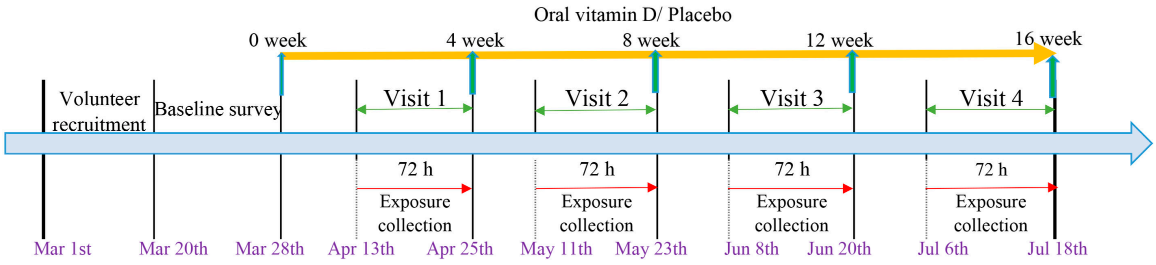

Figure S1 Follow-up protocol of the study.

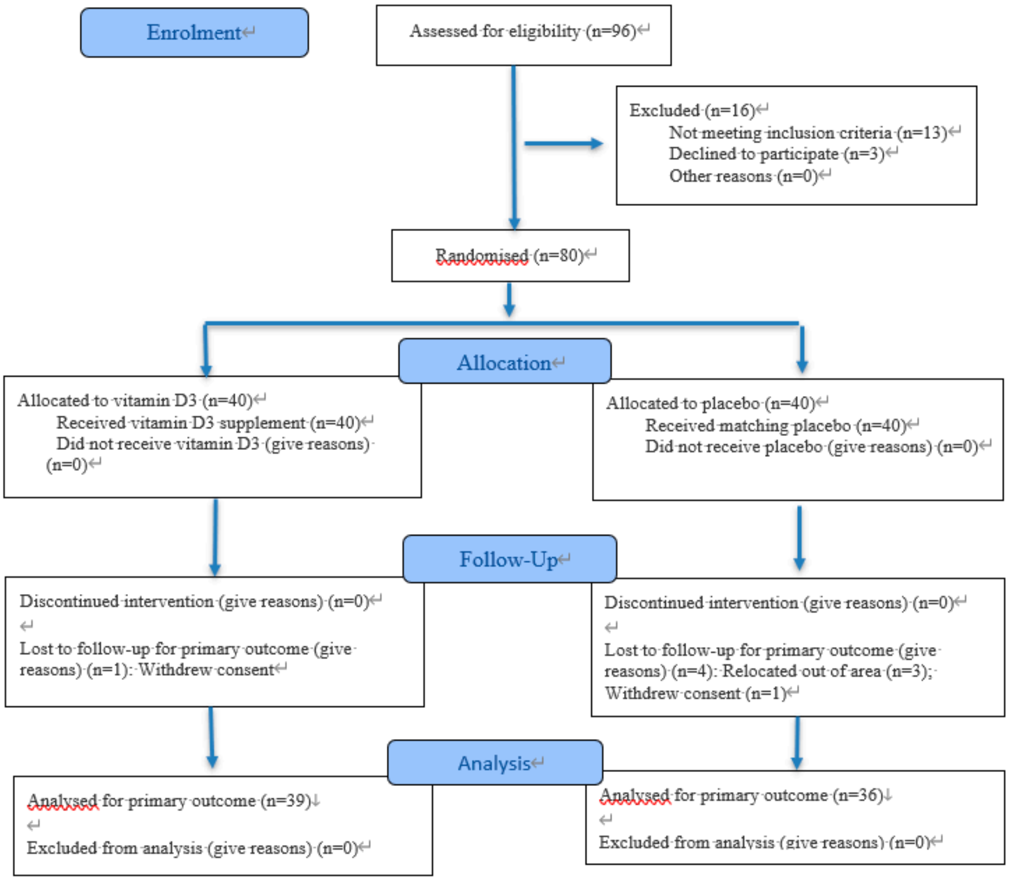

Figure S2 Participant flow through the trial

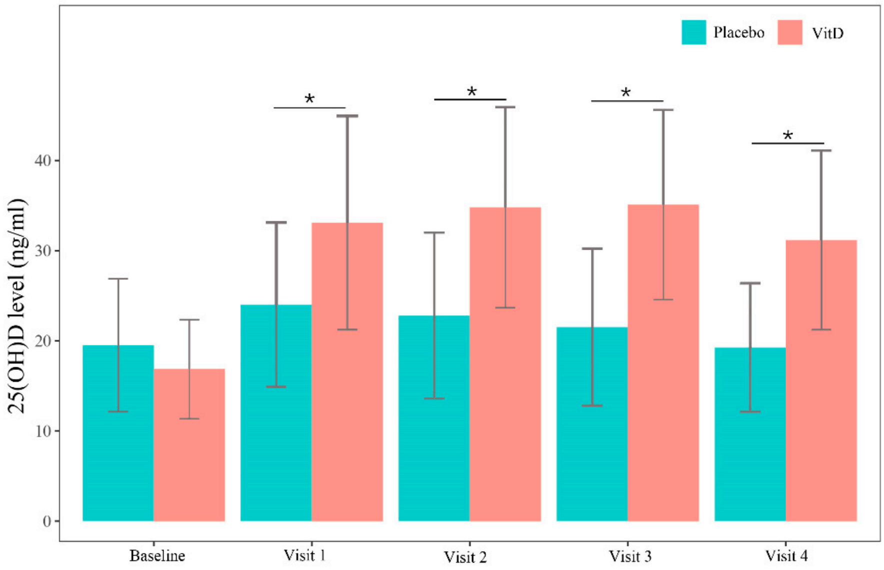

Figure S3 Distribution of serum 25(OH)D concentrations in the two study groups during the follow-up period

Abbreviation: 25(OH)D, 25-hydroxyvitamin D. *indicates a statistically significant difference between the two groups, P <0.05.

Table S1 Levels of lymphocyte counts during follow-up of study subjects.

Group	Cell counts	Visit 1	Visit 2	Visit 3	Visit 4	Mean levels of four follow-up visits
Vitamin D	CD3+T, cell/μL	1372.79 ± 466.17	1380.90 ± 479.63	1320.49 ± 498.07	1425.83 ± 493.60	1373.11 ± 479.85
	CD4+T, cell/μL	544.70 ± 192.83	571.44 ± 205.15	554.05 ± 212.00	589.75 ± 181.02	563.65 ± 197.14
	CD8+T, cell/μL	703.70 ± 374.99	679.64 ± 368.92	655.84 ± 356.09	730.67 ± 376.22	691.32 ± 365.95
	CD19+B, cell/μL	223.27 ± 107.71	251.08 ± 127.50	232.25 ± 108.14	240.95 ± 129.38	236.45 ± 117.15
	NK, cell/μL	329.49 ± 185.35	350.91 ± 209.52	361.93 ± 289.50	384.48 ± 348.23	355.11 ± 258.90
	LY, cell/μL	1957.06 ± 574.86	2013.87 ± 652.38	1944.05 ± 755.93	2073.69 ± 833.73	1993.52 ± 697.38
Placebo	CD3+T, cell/μL	1361.50 ± 488.10	1313.36 ± 438.74	1445.30 ± 473.77	1433.61 ± 463.04	1389.96 ± 465.16
	CD4+T, cell/μL	537.55 ± 196.27	524.16 ± 194.72	573.88 ± 213.78	577.98 ± 196.89	553.85 ± 199.72
	CD8+T, cell/μL	720.97 ± 342.77	690.18 ± 303.44	772.01 ± 334.44	763.85 ± 329.18	737.71 ± 326.62
	CD19+B, cell/μL	197.04 ± 98.40	186.30 ± 95.67	212.55 ± 98.09	210.17 ± 101.65	201.84 ± 97.96
	NK, cell/μL	373.82 ± 227.98	326.45 ± 176.65	382.64 ± 227.97	378.00 ± 207.50	366.43 ± 211.06
	LY, cell/μL	1969.86 ± 696.69	1868.78 ± 607.85	2071.37 ± 642.31	2053.31 ± 636.47	1993.72 ± 646.04

Note：CD3+T, total T lymphocytes; CD4+T, helper T lymphocytes; CD8+T, suppressor T lymphocytes; CD19+B, total B lymphocytes; NK, natural killer cells; LY, lymphocytes. Cell counts are presented as mean ± standard deviation.

Table S2 Distribution of immunomodulatory factors in subjects under the low and high PM exposure visit.

Immunomodulatory factors	Visit during lowest levels of PM exposure		Visit during Highest levels of PM exposure	
	Vitamin D group	Placebo group	Vitamin D group	Placebo group
TLR4, ng/mL	0.93 ± 0.48*	1.68 ± 1.10	0.88 ± 0.33*	1.95 ± 1.06
ICAM-1, ng/mL	379.51 ± 258.44	525.61 ± 402.81	398.66 ± 260.99	468.02 ± 341.74
IL-6, pg/mL	21.97 ± 7.24*	36.09 ± 14.37	23.31 ± 8.91	29.13 ± 10.05
TNF-α, pg/mL	35.20 ± 16.04	44.44 ± 12.57	41.44 ± 12.28	47.41 ± 13.90
IFNα2, pg/mL	53.38 ± 67.42	33.66 ± 9.58	47.54 ± 37.03	34.79 ± 9.76

Note: TLR4, Toll-like receptor 4; ICAM-1, intercellular adhesion molecule-1; IL-6, interleukin-6; TNF-α, tumor necrosis factor-α; IFNα2, interferon α2. Immunomodulatory factor concentrations are presented as means ± standard deviations. This sample set is identical to the 850K DNA methylation dataset, with 16 participants per group, where one measurement was obtained at the visit with the lowest PM concentration and one at the visit with the highest PM concentration for each participant. *Denotes statistically significant differences compared to the placebo group ($P < 0.05$).

Table S3 PM-associated changes in CD4+T and CD19+B cell counts in the vitamin D and placebo groups with additional adjustment for baseline 25(OH)D.

**Outcome**	**PM**	**Exposure window (h)**	**Placebo % change (95% CI)**	**Vitamin D % change (95% CI)**	**Between-group difference (95% CI)**	***P*-value**	**FDR adjust *P*-value**
CD4+T	PM_1	0–6	−2.74 (−5.10, −0.32)	−0.33 (−1.50, 0.85)	2.44 (−0.28, 5.17)	0.079	0.094
CD4+T	PM_1	7–12	−0.78 (−1.84, 0.30)	0.88 (−0.13, 1.89)	1.65 (0.19, 3.12)	0.027	0.049
CD4+T	PM_1	13–24	−1.52 (−3.05, 0.03)	0.48 (−1.15, 2.14)	2.02 (−0.24, 4.28)	0.080	0.094
CD4+T	PM_1	25–48	−1.75 (−3.25, −0.24)	0.07 (−1.34, 1.50)	1.84 (−0.25, 3.93)	0.084	0.094
CD4+T	PM_1	49–72	−1.52 (−3.65, 0.64)	0.57 (−0.88, 2.04)	2.10 (−0.51, 4.72)	0.115	0.115
CD4+T	PM_1	0–12	−1.38 (−2.93, 0.19)	0.47 (−0.73, 1.68)	1.86 (−0.12, 3.84)	0.066	0.089
CD4+T	PM_1	0–24	−2.08 (−3.93, −0.20)	0.77 (−0.88, 2.45)	2.87 (0.35, 5.40)	0.026	0.049
CD4+T	PM_1	0–48	−2.52 (−4.31, −0.70)	0.25 (−1.55, 2.07)	2.80 (0.21, 5.38)	0.034	0.056
CD4+T	PM_1	0–72	−2.66 (−4.70, −0.57)	0.54 (−1.27, 2.39)	3.23 (0.43, 6.03)	0.024	0.049
CD4+T	$PM_{2.5}$	0–6	−2.00 (−3.61, −0.36)	−0.07 (−0.85, 0.72)	1.95 (0.11, 3.78)	0.037	0.056
CD4+T	$PM_{2.5}$	7–12	−0.56 (−1.25, 0.14)	0.58 (0.02, 1.14)	1.13 (0.24, 2.03)	0.013	0.043
CD4+T	$PM_{2.5}$	13–24	−0.82 (−1.67, 0.04)	0.27 (−0.68, 1.22)	1.09 (−0.19, 2.37)	0.094	0.098
CD4+T	$PM_{2.5}$	25–48	−1.06 (−1.97, −0.14)	0.26 (−0.29, 0.82)	1.32 (0.25, 2.40)	0.016	0.043
CD4+T	$PM_{2.5}$	49–72	−0.92 (−2.19, 0.37)	0.52 (−0.28, 1.32)	1.44 (−0.08, 2.96)	0.063	0.089
CD4+T	$PM_{2.5}$	0–12	−1.00 (−2.02, 0.03)	0.44 (−0.28, 1.16)	1.44 (0.18, 2.70)	0.025	0.049
CD4+T	$PM_{2.5}$	0–24	−1.28 (−2.39, −0.16)	0.60 (−0.37, 1.59)	1.89 (0.40, 3.38)	0.013	0.043
CD4+T	$PM_{2.5}$	0–48	−1.49 (−2.56, −0.40)	0.42 (−0.40, 1.24)	1.91 (0.55, 3.28)	0.006	0.041
CD4+T	$PM_{2.5}$	0–72	−1.58 (−2.80, −0.34)	0.56 (−0.33, 1.46)	2.15 (0.62, 3.68)	0.006	0.041

CD4+T	PM_{10}	0–6	−1.75 (−3.15, −0.33)	−0.04 (−0.75, 0.68)	1.72 (0.12, 3.33)	0.035	0.056
CD4+T	PM_{10}	7–12	−0.53 (−1.16, 0.11)	0.54 (0.04, 1.05)	1.07 (0.26, 1.89)	0.010	0.043
CD4+T	PM_{10}	13–24	−0.74 (−1.49, 0.01)	0.24 (−0.61, 1.09)	0.98 (−0.16, 2.12)	0.091	0.098
CD4+T	PM_{10}	25–48	−0.95 (−1.78, −0.12)	0.23 (−0.23, 0.69)	1.19 (0.24, 2.14)	0.014	0.043
CD4+T	PM_{10}	49–72	−0.79 (−1.90, 0.35)	0.45 (−0.26, 1.16)	1.24 (−0.10, 2.57)	0.070	0.090
CD4+T	PM_{10}	0–12	−0.94 (−1.87, 0.00)	0.43 (−0.22, 1.08)	1.37 (0.22, 2.51)	0.019	0.047
CD4+T	PM_{10}	0–24	−1.15 (−2.13, −0.17)	0.58 (−0.30, 1.47)	1.74 (0.41, 3.07)	0.010	0.043
CD4+T	PM_{10}	0–48	−1.34 (−2.30, −0.38)	0.39 (−0.31, 1.09)	1.73 (0.54, 2.93)	0.005	0.041
CD4+T	PM_{10}	0–72	−1.39 (−2.48, −0.30)	0.50 (−0.27, 1.28)	1.90 (0.56, 3.25)	0.006	0.041
CD19+B	PM_1	0–6	−1.57 (−4.74, 1.70)	−0.45 (−1.86, 0.99)	1.14 (−2.43, 4.70)	0.533	0.553
CD19+B	PM_1	7–12	0.00 (−1.40, 1.42)	0.61 (−0.62, 1.86)	0.61 (−1.26, 2.49)	0.522	0.553
CD19+B	PM_1	13–24	−1.76 (−3.74, 0.26)	1.00 (−0.94, 2.99)	2.77 (−0.04, 5.59)	0.054	0.111
CD19+B	PM_1	25–48	−2.09 (−4.00, −0.15)	1.18 (−0.51, 2.90)	3.29 (0.70, 5.88)	0.013	0.032
CD19+B	PM_1	49–72	−3.01 (−5.61, −0.35)	1.04 (−0.67, 2.78)	4.09 (0.89, 7.30)	0.012	0.032
CD19+B	PM_1	0–12	−0.26 (−2.32, 1.84)	0.14 (−1.33, 1.63)	0.39 (−2.16, 2.95)	0.763	0.763
CD19+B	PM_1	0–24	−1.48 (−3.96, 1.05)	0.55 (−1.45, 2.58)	2.04 (−1.20, 5.28)	0.217	0.315
CD19+B	PM_1	0–48	−2.33 (−4.72, 0.11)	1.04 (−1.16, 3.29)	3.40 (0.09, 6.71)	0.044	0.099
CD19+B	PM_1	0–72	−3.01 (−5.68, −0.26)	1.52 (−0.68, 3.77)	4.56 (1.02, 8.11)	0.012	0.032
CD19+B	$PM_{2.5}$	0–6	−1.31 (−3.46, 0.90)	−0.34 (−1.28, 0.61)	0.97 (−1.43, 3.38)	0.428	0.502
CD19+B	$PM_{2.5}$	7–12	−0.20 (−1.11, 0.72)	0.45 (−0.24, 1.14)	0.65 (−0.50, 1.79)	0.269	0.363
CD19+B	$PM_{2.5}$	13–24	−0.90 (−2.01, 0.22)	0.44 (−0.69, 1.57)	1.34 (−0.25, 2.93)	0.099	0.190
CD19+B	$PM_{2.5}$	25–48	−1.26 (−2.43, −0.08)	0.72 (0.08, 1.37)	1.99 (0.64, 3.35)	0.004	0.027

CD19+B	$PM_{2.5}$	49–72	−1.88 (−3.44, −0.29)	0.74 (−0.20, 1.69)	2.64 (0.78, 4.50)	0.005	0.029
CD19+B	$PM_{2.5}$	0–12	−0.43 (−1.79, 0.95)	0.18 (−0.71, 1.08)	0.61 (−1.02, 2.25)	0.464	0.522
CD19+B	$PM_{2.5}$	0–24	−1.03 (−2.51, 0.46)	0.34 (−0.85, 1.54)	1.38 (−0.54, 3.29)	0.159	0.253
CD19+B	$PM_{2.5}$	0–48	−1.43 (−2.86, 0.02)	0.82 (−0.17, 1.82)	2.26 (0.50, 4.02)	0.012	0.032
CD19+B	$PM_{2.5}$	0–72	−1.84 (−3.44, −0.22)	1.06 (0.00, 2.14)	2.92 (0.97, 4.87)	0.003	0.027
CD19+B	PM_{10}	0–6	−1.21 (−3.08, 0.70)	−0.34 (−1.19, 0.53)	0.88 (−1.22, 2.98)	0.412	0.502
CD19+B	PM_{10}	7–12	−0.23 (−1.07, 0.61)	0.42 (−0.20, 1.05)	0.65 (−0.39, 1.70)	0.222	0.315
CD19+B	PM_{10}	13–24	−0.84 (−1.82, 0.15)	0.32 (−0.69, 1.34)	1.16 (−0.26, 2.57)	0.108	0.195
CD19+B	PM_{10}	25–48	−1.16 (−2.22, −0.09)	0.60 (0.07, 1.14)	1.77 (0.57, 2.97)	0.004	0.027
CD19+B	PM_{10}	49–72	−1.63 (−3.01, −0.24)	0.61 (−0.22, 1.46)	2.26 (0.62, 3.89)	0.007	0.031
CD19+B	PM_{10}	0–12	−0.48 (−1.71, 0.77)	0.16 (−0.65, 0.97)	0.63 (−0.85, 2.12)	0.403	0.502
CD19+B	PM_{10}	0–24	−1.00 (−2.30, 0.32)	0.26 (−0.81, 1.35)	1.26 (−0.44, 2.97)	0.147	0.248
CD19+B	PM_{10}	0–48	−1.33 (−2.60, −0.04)	0.71 (−0.13, 1.56)	2.05 (0.50, 3.60)	0.009	0.032
CD19+B	PM_{10}	0–72	−1.67 (−3.08, −0.23)	0.91 (−0.01, 1.84)	2.59 (0.87, 4.30)	0.003	0.027

Note: Models were adjusted for age, sex, BMI, work type, family income, education level, marital status, physical ac-tivity, dietary habits, drinking frequency, emotional disorder, sleep disorder, temperature, relative humidity, and baseline 25(OH)D.

Table S4 PM-associated changes in CD4+T and CD19+B cell counts in the vitamin D and placebo groups with additional adjustment for the corresponding baseline immune cell count.

Outcome	PM	Exposure window (h)	Placebo % change (95% CI)	Vitamin D % change (95% CI)	Between-group difference (95% CI)	*P*-value	FDR adjust *P*-value
CD4+T	PM_1	0–6	−2.34 (−4.64, 0.01)	−0.18 (−1.30, 0.95)	2.19 (−0.44, 4.83)	0.103	0.103
CD4+T	PM_1	7–12	−0.65 (−1.67, 0.39)	1.14 (0.20, 2.08)	1.78 (0.38, 3.17)	0.012	0.023
CD4+T	PM_1	13–24	−1.52 (−3.02, −0.01)	0.99 (−0.53, 2.53)	2.52 (0.37, 4.67)	0.022	0.034
CD4+T	PM_1	25–48	−1.63 (−3.08, −0.15)	0.47 (−0.87, 1.83)	2.11 (0.10, 4.11)	0.039	0.052
CD4+T	PM_1	49–72	−1.47 (−3.53, 0.63)	0.75 (−0.62, 2.14)	2.23 (−0.28, 4.74)	0.082	0.085
CD4+T	PM_1	0–12	−1.18 (−2.68, 0.34)	0.77 (−0.36, 1.92)	1.96 (0.06, 3.86)	0.044	0.053
CD4+T	PM_1	0–24	−1.91 (−3.71, −0.09)	1.26 (−0.28, 2.83)	3.19 (0.79, 5.59)	0.009	0.019
CD4+T	PM_1	0–48	−2.36 (−4.09, −0.59)	0.94 (−0.73, 2.64)	3.32 (0.88, 5.77)	0.008	0.019
CD4+T	PM_1	0–72	−2.45 (−4.43, −0.43)	1.06 (−0.62, 2.76)	3.53 (0.88, 6.17)	0.009	0.019
CD4+T	$PM_{2.5}$	0–6	−1.74 (−3.31, −0.15)	0.05 (−0.69, 0.80)	1.81 (0.04, 3.59)	0.045	0.053
CD4+T	$PM_{2.5}$	7–12	−0.48 (−1.15, 0.19)	0.72 (0.20, 1.25)	1.20 (0.35, 2.05)	0.006	0.019
CD4+T	$PM_{2.5}$	13–24	−0.81 (−1.64, 0.03)	0.55 (−0.33, 1.43)	1.36 (0.14, 2.58)	0.028	0.041
CD4+T	$PM_{2.5}$	25–48	−0.98 (−1.87, −0.09)	0.42 (−0.11, 0.95)	1.41 (0.36, 2.45)	0.008	0.019
CD4+T	$PM_{2.5}$	49–72	−0.91 (−2.14, 0.34)	0.61 (−0.14, 1.37)	1.52 (0.07, 2.98)	0.041	0.052
CD4+T	$PM_{2.5}$	0–12	−0.87 (−1.87, 0.13)	0.63 (−0.05, 1.31)	1.51 (0.30, 2.72)	0.015	0.025
CD4+T	$PM_{2.5}$	0–24	−1.19 (−2.27, −0.10)	0.89 (−0.01, 1.81)	2.09 (0.67, 3.51)	0.004	0.018
CD4+T	$PM_{2.5}$	0–48	−1.40 (−2.44, −0.35)	0.71 (−0.06, 1.49)	2.12 (0.81, 3.43)	0.002	0.015

CD4+T	$PM_{2.5}$	0–72	−1.47 (−2.66, −0.28)	0.81 (−0.02, 1.64)	2.29 (0.83, 3.75)	0.002	0.015
CD4+T	PM_{10}	0–6	−1.47 (−2.83, −0.09)	0.07 (−0.61, 0.75)	1.55 (0.00, 3.10)	0.050	0.054
CD4+T	PM_{10}	7–12	−0.44 (−1.05, 0.17)	0.67 (0.20, 1.15)	1.11 (0.34, 1.89)	0.005	0.019
CD4+T	PM_{10}	13–24	−0.73 (−1.46, 0.01)	0.48 (−0.31, 1.28)	1.21 (0.12, 2.30)	0.029	0.041
CD4+T	PM_{10}	25–48	−0.89 (−1.69, −0.08)	0.35 (−0.09, 0.80)	1.25 (0.32, 2.17)	0.008	0.019
CD4+T	PM_{10}	49–72	−0.76 (−1.85, 0.34)	0.53 (−0.14, 1.21)	1.30 (0.01, 2.58)	0.048	0.054
CD4+T	PM_{10}	0–12	−0.80 (−1.70, 0.11)	0.59 (−0.02, 1.21)	1.39 (0.30, 2.49)	0.013	0.023
CD4+T	PM_{10}	0–24	−1.06 (−2.01, −0.10)	0.84 (0.01, 1.67)	1.90 (0.63, 3.16)	0.003	0.018
CD4+T	PM_{10}	0–48	−1.26 (−2.19, −0.32)	0.63 (−0.03, 1.30)	1.89 (0.74, 3.05)	0.001	0.015
CD4+T	PM_{10}	0–72	−1.29 (−2.34, −0.23)	0.71 (0.00, 1.44)	2.01 (0.72, 3.30)	0.002	0.015
CD19+B	PM_1	0–6	−1.13 (−4.23, 2.07)	−0.26 (−1.63, 1.13)	0.88 (−2.59, 4.35)	0.620	0.644
CD19+B	PM_1	7–12	0.05 (−1.32, 1.43)	0.78 (−0.40, 1.97)	0.73 (−1.08, 2.54)	0.432	0.507
CD19+B	PM_1	13–24	−1.70 (−3.65, 0.30)	1.36 (−0.49, 3.25)	3.07 (0.34, 5.79)	0.027	0.057
CD19+B	PM_1	25–48	−1.98 (−3.87, −0.05)	1.45 (−0.18, 3.10)	3.43 (0.90, 5.96)	0.008	0.019
CD19+B	PM_1	49–72	−3.02 (−5.57, −0.40)	1.29 (−0.38, 2.98)	4.34 (1.20, 7.48)	0.007	0.019
CD19+B	PM_1	0–12	−0.12 (−2.13, 1.93)	0.38 (−1.04, 1.81)	0.50 (−1.98, 2.97)	0.695	0.695
CD19+B	PM_1	0–24	−1.30 (−3.72, 1.18)	0.96 (−0.95, 2.89)	2.26 (−0.87, 5.38)	0.157	0.235
CD19+B	PM_1	0–48	−2.13 (−4.47, 0.27)	1.55 (−0.52, 3.67)	3.69 (0.51, 6.87)	0.023	0.052
CD19+B	PM_1	0–72	−2.77 (−5.39, −0.07)	1.97 (−0.11, 4.08)	4.75 (1.33, 8.17)	0.006	0.019
CD19+B	$PM_{2.5}$	0–6	−1.00 (−3.11, 1.15)	−0.21 (−1.12, 0.71)	0.79 (−1.55, 3.13)	0.506	0.567
CD19+B	$PM_{2.5}$	7–12	−0.16 (−1.05, 0.73)	0.54 (−0.12, 1.20)	0.70 (−0.41, 1.80)	0.219	0.295
CD19+B	$PM_{2.5}$	13–24	−0.89 (−1.98, 0.22)	0.66 (−0.41, 1.74)	1.55 (0.01, 3.09)	0.049	0.094

CD19+B	$PM_{2.5}$	25–48	−1.21 (−2.36, −0.04)	0.81 (0.18, 1.45)	2.02 (0.69, 3.36)	0.003	0.017
CD19+B	$PM_{2.5}$	49–72	−1.92 (−3.46, −0.36)	0.87 (−0.04, 1.80)	2.81 (0.99, 4.64)	0.003	0.017
CD19+B	$PM_{2.5}$	0–12	−0.34 (−1.66, 1.00)	0.32 (−0.53, 1.18)	0.65 (−0.93, 2.24)	0.419	0.507
CD19+B	$PM_{2.5}$	0–24	−0.95 (−2.40, 0.52)	0.58 (−0.54, 1.72)	1.53 (−0.32, 3.39)	0.104	0.166
CD19+B	$PM_{2.5}$	0–48	−1.33 (−2.74, 0.09)	1.02 (0.07, 1.97)	2.35 (0.64, 4.07)	0.007	0.019
CD19+B	$PM_{2.5}$	0–72	−1.74 (−3.31, −0.15)	1.26 (0.25, 2.28)	3.01 (1.11, 4.90)	0.002	0.017
CD19+B	PM_{10}	0–6	−0.88 (−2.71, 0.99)	−0.22 (−1.05, 0.61)	0.66 (−1.38, 2.70)	0.525	0.567
CD19+B	PM_{10}	7–12	−0.19 (−1.00, 0.63)	0.49 (−0.11, 1.09)	0.68 (−0.33, 1.69)	0.187	0.266
CD19+B	PM_{10}	13–24	−0.83 (−1.79, 0.15)	0.51 (−0.45, 1.48)	1.34 (−0.03, 2.71)	0.055	0.100
CD19+B	PM_{10}	25–48	−1.10 (−2.14, −0.04)	0.68 (0.15, 1.20)	1.78 (0.59, 2.96)	0.003	0.017
CD19+B	PM_{10}	49–72	−1.64 (−2.99, −0.26)	0.73 (−0.09, 1.55)	2.37 (0.77, 3.98)	0.004	0.017
CD19+B	PM_{10}	0–12	−0.36 (−1.56, 0.85)	0.27 (−0.50, 1.05)	0.64 (−0.80, 2.07)	0.384	0.494
CD19+B	PM_{10}	0–24	−0.91 (−2.18, 0.39)	0.47 (−0.55, 1.50)	1.38 (−0.27, 3.03)	0.100	0.166
CD19+B	PM_{10}	0–48	−1.22 (−2.48, 0.04)	0.87 (0.06, 1.69)	2.10 (0.59, 3.61)	0.006	0.019
CD19+B	PM_{10}	0–72	−1.56 (−2.95, −0.14)	1.07 (0.19, 1.96)	2.63 (0.97, 4.30)	0.002	0.017

Note: Models were adjusted for age, sex, BMI, work type, family income, education level, marital status, physical ac-tivity, dietary habits, drinking frequency, emotional disorder, sleep disorder, temperature, relative humidity, and corresponding baseline immune cell count.

Table S5 PM-associated changes in CD4+T and CD19+B cell counts in the vitamin D and placebo groups with additional adjustment for baseline 25(OH)D and the corresponding baseline immune cell count.

**Outcome**	**PM**	**Exposure window (h)**	**Placebo % change (95% CI)**	**Vitamin D % change (95% CI)**	**Between-group difference (95% CI)**	***P*-value**	**FDR adjust *P*-value**
CD4+T	PM_1	0–6	−2.39 (−4.70, −0.03)	−0.13 (−1.25, 1.00)	2.29 (−0.35, 4.93)	0.089	0.093
CD4+T	PM_1	7–12	−0.65 (−1.68, 0.39)	1.09 (0.15, 2.04)	1.74 (0.34, 3.14)	0.015	0.025
CD4+T	PM_1	13–24	−1.52 (−3.02, 0.00)	0.78 (−0.76, 2.35)	2.31 (0.14, 4.49)	0.037	0.058
CD4+T	PM_1	25–48	−1.64 (−3.10, −0.16)	0.32 (−1.04, 1.69)	1.97 (−0.04, 3.99)	0.055	0.062
CD4+T	PM_1	49–72	−1.47 (−3.52, 0.64)	0.61 (−0.78, 2.01)	2.08 (−0.45, 4.61)	0.106	0.106
CD4+T	PM_1	0–12	−1.19 (−2.70, 0.34)	0.77 (−0.36, 1.91)	1.96 (0.06, 3.86)	0.043	0.058
CD4+T	PM_1	0–24	−1.92 (−3.72, −0.09)	1.15 (−0.39, 2.72)	3.08 (0.68, 5.49)	0.012	0.025
CD4+T	PM_1	0–48	−2.37 (−4.11, −0.61)	0.76 (−0.93, 2.47)	3.15 (0.69, 5.62)	0.012	0.025
CD4+T	PM_1	0–72	−2.46 (−4.45, −0.44)	0.85 (−0.85, 2.57)	3.34 (0.67, 6.00)	0.014	0.025
CD4+T	$PM_{2.5}$	0–6	−1.78 (−3.35, −0.18)	0.08 (−0.66, 0.83)	1.87 (0.10, 3.65)	0.039	0.058
CD4+T	$PM_{2.5}$	7–12	−0.48 (−1.15, 0.19)	0.70 (0.17, 1.22)	1.18 (0.32, 2.03)	0.007	0.023
CD4+T	$PM_{2.5}$	13–24	−0.81 (−1.64, 0.03)	0.43 (−0.46, 1.34)	1.24 (0.01, 2.47)	0.048	0.059
CD4+T	$PM_{2.5}$	25–48	−0.99 (−1.88, −0.09)	0.37 (−0.17, 0.91)	1.36 (0.32, 2.41)	0.011	0.025
CD4+T	$PM_{2.5}$	49–72	−0.90 (−2.14, 0.35)	0.54 (−0.23, 1.31)	1.44 (−0.03, 2.91)	0.054	0.062
CD4+T	$PM_{2.5}$	0–12	−0.88 (−1.88, 0.12)	0.62 (−0.06, 1.30)	1.50 (0.29, 2.71)	0.015	0.025
CD4+T	$PM_{2.5}$	0–24	−1.19 (−2.27, −0.10)	0.82 (−0.09, 1.74)	2.02 (0.60, 3.44)	0.005	0.022
CD4+T	$PM_{2.5}$	0–48	−1.41 (−2.45, −0.35)	0.64 (−0.14, 1.42)	2.05 (0.73, 3.37)	0.002	0.022

CD4+T	$PM_{2.5}$	0–72	−1.48 (−2.66, −0.28)	0.72 (−0.12, 1.56)	2.20 (0.73, 3.68)	0.003	0.022
CD4+T	PM_{10}	0–6	−1.50 (−2.87, −0.12)	0.09 (−0.58, 0.77)	1.61 (0.05, 3.16)	0.042	0.058
CD4+T	PM_{10}	7–12	−0.45 (−1.06, 0.17)	0.65 (0.17, 1.13)	1.10 (0.32, 1.87)	0.006	0.022
CD4+T	PM_{10}	13–24	−0.73 (−1.46, 0.02)	0.38 (−0.42, 1.19)	1.11 (0.01, 2.20)	0.048	0.059
CD4+T	PM_{10}	25–48	−0.90 (−1.70, −0.08)	0.32 (−0.13, 0.76)	1.22 (0.29, 2.14)	0.010	0.025
CD4+T	PM_{10}	49–72	−0.76 (−1.85, 0.34)	0.46 (−0.22, 1.15)	1.23 (−0.07, 2.52)	0.063	0.068
CD4+T	PM_{10}	0–12	−0.81 (−1.71, 0.10)	0.58 (−0.03, 1.20)	1.39 (0.30, 2.49)	0.013	0.025
CD4+T	PM_{10}	0–24	−1.06 (−2.02, −0.10)	0.77 (−0.05, 1.61)	1.84 (0.57, 3.11)	0.005	0.022
CD4+T	PM_{10}	0–48	−1.26 (−2.20, −0.33)	0.57 (−0.10, 1.24)	1.84 (0.68, 3.00)	0.002	0.022
CD4+T	PM_{10}	0–72	−1.30 (−2.35, −0.23)	0.64 (−0.09, 1.37)	1.94 (0.65, 3.24)	0.003	0.022
CD19+B	PM_1	0–6	−1.18 (−4.28, 2.02)	−0.17 (−1.54, 1.22)	1.01 (−2.46, 4.49)	0.567	0.589
CD19+B	PM_1	7–12	0.03 (−1.34, 1.42)	0.75 (−0.43, 1.93)	0.71 (−1.10, 2.52)	0.440	0.517
CD19+B	PM_1	13–24	−1.69 (−3.65, 0.30)	1.13 (−0.74, 3.03)	2.83 (0.09, 5.57)	0.043	0.089
CD19+B	PM_1	25–48	−2.00 (−3.89, −0.07)	1.29 (−0.34, 2.95)	3.30 (0.77, 5.84)	0.011	0.026
CD19+B	PM_1	49–72	−3.05 (−5.60, −0.43)	1.14 (−0.54, 2.84)	4.23 (1.08, 7.38)	0.008	0.024
CD19+B	PM_1	0–12	−0.15 (−2.16, 1.91)	0.40 (−1.01, 1.83)	0.55 (−1.93, 3.02)	0.666	0.666
CD19+B	PM_1	0–24	−1.32 (−3.74, 1.16)	0.86 (−1.03, 2.80)	2.19 (−0.93, 5.31)	0.170	0.254
CD19+B	PM_1	0–48	−2.16 (−4.50, 0.24)	1.37 (−0.71, 3.48)	3.54 (0.35, 6.72)	0.029	0.066
CD19+B	PM_1	0–72	−2.81 (−5.44, −0.12)	1.74 (−0.35, 3.86)	4.57 (1.14, 8.00)	0.009	0.024
CD19+B	$PM_{2.5}$	0–6	−1.04 (−3.15, 1.12)	−0.16 (−1.07, 0.76)	0.88 (−1.46, 3.22)	0.461	0.517
CD19+B	$PM_{2.5}$	7–12	−0.17 (−1.06, 0.72)	0.52 (−0.14, 1.18)	0.69 (−0.42, 1.80)	0.225	0.304
CD19+B	$PM_{2.5}$	13–24	−0.88 (−1.98, 0.22)	0.53 (−0.55, 1.62)	1.41 (−0.13, 2.96)	0.074	0.142

CD19+B	$PM_{2.5}$	25–48	−1.22 (−2.38, −0.05)	0.77 (0.13, 1.40)	1.99 (0.65, 3.33)	0.004	0.020
CD19+B	$PM_{2.5}$	49–72	−1.94 (−3.48, −0.38)	0.79 (−0.13, 1.72)	2.75 (0.92, 4.57)	0.003	0.020
CD19+B	$PM_{2.5}$	0–12	−0.35 (−1.68, 0.99)	0.32 (−0.52, 1.18)	0.68 (−0.91, 2.26)	0.403	0.494
CD19+B	$PM_{2.5}$	0–24	−0.96 (−2.40, 0.51)	0.52 (−0.60, 1.66)	1.48 (−0.37, 3.33)	0.117	0.186
CD19+B	$PM_{2.5}$	0–48	−1.35 (−2.75, 0.08)	0.94 (0.00, 1.90)	2.29 (0.58, 4.01)	0.009	0.024
CD19+B	$PM_{2.5}$	0–72	−1.76 (−3.33, −0.17)	1.15 (0.14, 2.18)	2.93 (1.03, 4.83)	0.003	0.020
CD19+B	PM_{10}	0–6	−0.91 (−2.74, 0.96)	−0.18 (−1.00, 0.65)	0.74 (−1.30, 2.78)	0.479	0.517
CD19+B	PM_{10}	7–12	−0.20 (−1.01, 0.62)	0.47 (−0.12, 1.08)	0.67 (−0.34, 1.69)	0.191	0.272
CD19+B	PM_{10}	13–24	−0.82 (−1.79, 0.15)	0.40 (−0.58, 1.37)	1.22 (−0.16, 2.60)	0.083	0.149
CD19+B	PM_{10}	25–48	−1.11 (−2.16, −0.05)	0.64 (0.12, 1.16)	1.75 (0.57, 2.94)	0.004	0.020
CD19+B	PM_{10}	49–72	−1.65 (−3.01, −0.28)	0.65 (−0.17, 1.48)	2.32 (0.71, 3.93)	0.005	0.022
CD19+B	PM_{10}	0–12	−0.38 (−1.58, 0.83)	0.28 (−0.49, 1.05)	0.66 (−0.78, 2.10)	0.367	0.472
CD19+B	PM_{10}	0–24	−0.91 (−2.19, 0.38)	0.42 (−0.60, 1.45)	1.34 (−0.31, 2.99)	0.112	0.186
CD19+B	PM_{10}	0–48	−1.24 (−2.49, 0.03)	0.81 (0.00, 1.63)	2.05 (0.55, 3.56)	0.008	0.024
CD19+B	PM_{10}	0–72	−1.58 (−2.97, −0.16)	0.99 (0.10, 1.88)	2.57 (0.90, 4.24)	0.003	0.020

Note: Models were adjusted for age, sex, BMI, work type, family income, education level, marital status, physical ac-tivity, dietary habits, drinking frequency, emotional disorder, sleep disorder, temperature, relative humidity, baseline 25(OH)D, and corresponding baseline immune cell count.

Table S6 PM-associated changes in CD4+T and CD19+B cell counts and PM-by-treatment interactions.

Outcome	PM	Exposure window (h)	Placebo % change (95% CI)	Vitamin D % change (95% CI)	Interaction effect % (95% CI)	*P* for interaction	FDR adjust *P*-value
CD4+T	PM_1	0–6	−1.78 (−4.38, 0.89)	−0.20 (−1.30, 0.92)	1.61 (−1.24, 4.55)	0.270	0.317
CD4+T	PM_1	7–12	−0.12 (−1.26, 1.03)	0.94 (−0.03, 1.92)	1.06 (−0.43, 2.57)	0.163	0.213
CD4+T	PM_1	13–24	−0.58 (−2.29, 1.16)	0.30 (−1.18, 1.79)	0.89 (−1.27, 3.09)	0.421	0.426
CD4+T	PM_1	25–48	−1.37 (−3.03, 0.31)	0.15 (−1.10, 1.42)	1.54 (−0.49, 3.62)	0.138	0.213
CD4+T	PM_1	49–72	−1.29 (−3.48, 0.95)	0.57 (−0.73, 1.88)	1.88 (−0.62, 4.44)	0.141	0.213
CD4+T	PM_1	0–12	−0.53 (−2.20, 1.17)	0.55 (−0.59, 1.71)	1.09 (−0.93, 3.14)	0.291	0.327
CD4+T	PM_1	0–24	−0.74 (−2.78, 1.35)	0.74 (−0.79, 2.29)	1.49 (−1.00, 4.04)	0.241	0.296
CD4+T	PM_1	0–48	−1.51 (−3.50, 0.51)	0.40 (−1.21, 2.05)	1.95 (−0.55, 4.51)	0.126	0.213
CD4+T	PM_1	0–72	−1.50 (−3.73, 0.79)	0.55 (−1.08, 2.21)	2.08 (−0.61, 4.85)	0.129	0.213
CD4+T	$PM_{2.5}$	0–6	−1.33 (−3.11, 0.49)	0.04 (−0.69, 0.78)	1.39 (−0.55, 3.36)	0.160	0.213
CD4+T	$PM_{2.5}$	7–12	−0.11 (−0.85, 0.63)	0.61 (0.07, 1.16)	0.73 (−0.19, 1.65)	0.119	0.213
CD4+T	$PM_{2.5}$	13–24	−0.36 (−1.32, 0.61)	0.15 (−0.71, 1.01)	0.51 (−0.72, 1.76)	0.416	0.426
CD4+T	$PM_{2.5}$	25–48	−0.80 (−1.81, 0.22)	0.36 (−0.14, 0.86)	1.17 (0.04, 2.30)	**0.042**	0.213
CD4+T	$PM_{2.5}$	49–72	−0.79 (−2.12, 0.55)	0.54 (−0.17, 1.26)	1.34 (−0.14, 2.84)	0.075	0.213
CD4+T	$PM_{2.5}$	0–12	−0.40 (−1.50, 0.72)	0.51 (−0.18, 1.20)	0.91 (−0.38, 2.22)	0.166	0.213
CD4+T	$PM_{2.5}$	0–24	−0.50 (−1.73, 0.75)	0.58 (−0.33, 1.49)	1.08 (−0.41, 2.59)	0.154	0.213
CD4+T	$PM_{2.5}$	0–48	−0.89 (−2.08, 0.32)	0.55 (−0.20, 1.30)	1.45 (0.05, 2.86)	**0.042**	0.213
CD4+T	$PM_{2.5}$	0–72	−0.90 (−2.24, 0.46)	0.63 (−0.17, 1.44)	1.55 (0.01, 3.10)	**0.048**	0.213

CD4+T	PM_{10}	0–6	−1.23 (−2.77, 0.33)	0.05 (−0.61, 0.72)	1.30 (−0.38, 3.00)	0.129	0.213
CD4+T	PM_{10}	7–12	−0.12 (−0.79, 0.56)	0.57 (0.07, 1.06)	0.68 (−0.15, 1.52)	0.107	0.213
CD4+T	PM_{10}	13–24	−0.33 (−1.17, 0.52)	0.11 (−0.65, 0.88)	0.44 (−0.64, 1.54)	0.426	0.426
CD4+T	PM_{10}	25–48	−0.72 (−1.63, 0.20)	0.30 (−0.11, 0.71)	1.03 (0.04, 2.03)	0.042	0.213
CD4+T	PM_{10}	49–72	−0.71 (−1.87, 0.46)	0.45 (−0.18, 1.09)	1.17 (−0.12, 2.48)	0.076	0.213
CD4+T	PM_{10}	0–12	−0.41 (−1.41, 0.60)	0.47 (−0.15, 1.10)	0.89 (−0.28, 2.07)	0.136	0.213
CD4+T	PM_{10}	0–24	−0.48 (−1.56, 0.62)	0.52 (−0.30, 1.34)	1.00 (−0.32, 2.33)	0.136	0.213
CD4+T	PM_{10}	0–48	−0.80 (−1.86, 0.26)	0.48 (−0.16, 1.12)	1.29 (0.07, 2.53)	**0.038**	0.213
CD4+T	PM_{10}	0–72	−0.80 (−1.98, 0.39)	0.54 (−0.15, 1.24)	1.36 (0.02, 2.71)	**0.047**	0.213
CD19+B	PM_1	0–6	−1.81 (−4.94, 1.42)	−0.28 (−1.61, 1.07)	1.56 (−1.87, 5.12)	0.376	0.612
CD19+B	PM_1	7–12	0.31 (−1.07, 1.72)	0.60 (−0.57, 1.78)	0.29 (−1.50, 2.11)	0.753	0.781
CD19+B	PM_1	13–24	−0.77 (−2.82, 1.32)	0.28 (−1.48, 2.08)	1.06 (−1.53, 3.71)	0.424	0.612
CD19+B	PM_1	25–48	−1.51 (−3.47, 0.50)	1.05 (−0.45, 2.57)	2.60 (0.16, 5.10)	**0.037**	0.107
CD19+B	PM_1	49–72	−2.44 (−5.00, 0.19)	1.05 (−0.49, 2.62)	3.58 (0.57, 6.67)	**0.020**	0.075
CD19+B	PM_1	0–12	−0.06 (−2.09, 2.02)	0.25 (−1.12, 1.64)	0.31 (−2.11, 2.78)	0.804	0.804
CD19+B	PM_1	0–24	−0.61 (−3.08, 1.93)	0.39 (−1.44, 2.26)	1.01 (−1.98, 4.09)	0.511	0.626
CD19+B	PM_1	0–48	−1.48 (−3.88, 0.98)	0.97 (−0.99, 2.97)	2.49 (−0.54, 5.60)	0.108	0.243
CD19+B	PM_1	0–72	−1.84 (−4.50, 0.89)	1.38 (−0.58, 3.37)	3.28 (0.03, 6.63)	**0.048**	0.117
CD19+B	$PM_{2.5}$	0–6	−1.43 (−3.58, 0.76)	−0.22 (−1.09, 0.67)	1.23 (−1.10, 3.62)	0.300	0.579
CD19+B	$PM_{2.5}$	7–12	0.06 (−0.84, 0.97)	0.45 (−0.21, 1.10)	0.38 (−0.72, 1.50)	0.496	0.626
CD19+B	$PM_{2.5}$	13–24	−0.42 (−1.57, 0.75)	0.04 (−0.99, 1.07)	0.46 (−1.02, 1.96)	0.546	0.626
CD19+B	$PM_{2.5}$	25–48	−0.90 (−2.10, 0.30)	0.74 (0.15, 1.33)	1.66 (0.32, 3.01)	**0.015**	0.074

CD19+B	$PM_{2.5}$	49–72	−1.56 (−3.11, 0.02)	0.78 (−0.07, 1.63)	2.37 (0.61, 4.17)	**0.009**	0.074
CD19+B	$PM_{2.5}$	0–12	−0.22 (−1.56, 1.14)	0.24 (−0.58, 1.08)	0.47 (−1.09, 2.05)	0.556	0.626
CD19+B	$PM_{2.5}$	0–24	−0.49 (−1.98, 1.02)	0.23 (−0.86, 1.33)	0.72 (−1.07, 2.55)	0.431	0.612
CD19+B	$PM_{2.5}$	0–48	−0.91 (−2.35, 0.56)	0.85 (−0.05, 1.76)	1.77 (0.08, 3.48)	**0.040**	0.107
CD19+B	$PM_{2.5}$	0–72	−1.17 (−2.76, 0.45)	1.05 (0.10, 2.02)	2.25 (0.41, 4.12)	**0.017**	0.074
CD19+B	PM_{10}	0–6	−1.43 (−3.28, 0.46)	−0.23 (−1.02, 0.57)	1.21 (−0.80, 3.27)	0.239	0.496
CD19+B	PM_{10}	7–12	0.00 (−0.82, 0.83)	0.41 (−0.18, 1.01)	0.41 (−0.60, 1.43)	0.423	0.612
CD19+B	PM_{10}	13–24	−0.40 (−1.41, 0.63)	−0.04 (−0.95, 0.89)	0.36 (−0.94, 1.68)	0.585	0.631
CD19+B	PM_{10}	25–48	−0.82 (−1.89, 0.26)	0.62 (0.13, 1.11)	1.45 (0.27, 2.64)	**0.016**	0.074
CD19+B	PM_{10}	49–72	−1.41 (−2.77, −0.03)	0.64 (−0.11, 1.40)	2.08 (0.54, 3.65)	**0.008**	0.074
CD19+B	PM_{10}	0–12	−0.32 (−1.53, 0.91)	0.21 (−0.54, 0.96)	0.53 (−0.88, 1.96)	0.461	0.622
CD19+B	PM_{10}	0–24	−0.53 (−1.85, 0.80)	0.15 (−0.83, 1.14)	0.69 (−0.90, 2.30)	0.397	0.612
CD19+B	PM_{10}	0–48	−0.85 (−2.12, 0.44)	0.73 (−0.04, 1.50)	1.59 (0.12, 3.09)	**0.034**	0.107
CD19+B	PM_{10}	0–72	−1.07 (−2.47, 0.35)	0.90 (0.07, 1.73)	1.99 (0.38, 3.62)	**0.015**	0.074

All models included PM, treatment group, their interaction, sex, age, BMI, marital status, occupation, household income, education, drinking frequency, physical activity, emotional disorder, sleep disorder, diet, and window-matched temperature and humidity natural splines.

Table S7 Association between Vitamin D Intervention and Whole-Genome DNA Methylation in people living with HIV.

CpG	Chromosome	Location	Gene	Gene region	β(95%CI)	*P*-value	FDR adjust *P*-value
cg02037503	14	23540729	ACIN1	Body	−0.18 (−0.24, −0.13)	4.75E-08	0.015
cg21938718	20	43378461	KCNK15	Body	0.31 (0.21, 0.40)	9.50E-08	0.015
cg00129557	13	33607618	KL	Body	−0.68 (−0.89, −0.47)	1.02E-07	0.015
cg13791558	3	14371041			−0.36 (−0.47, −0.25)	1.05E-07	0.015
cg04817034	11	77920577	USP35	Body	−0.29 (−0.38, −0.20)	1.06E-07	0.015
cg26346621	12	6873348			−0.28 (−0.37, −0.19)	1.43E-07	0.016
cg11667959	2	71291991			−0.28 (−0.37, −0.19)	1.56E-07	0.016
cg10276433	6	47011312	GPR110	TSS1500	−0.48 (−0.63, −0.33)	1.91E-07	0.016
cg12202498	3	15482605	EAF1	3'UTR	−0.36 (−0.47, −0.24)	1.97E-07	0.016
cg12210036	1	204183581	GOLT1A	TSS1500	−0.38 (−0.51, −0.26)	2.97E-07	0.020
cg07324393	4	74740320			−0.41 (−0.54, −0.27)	3.10E-07	0.020
cg17961106	14	54969431			−0.33 (−0.44, −0.22)	3.49E-07	0.020
cg04969406	22	37977425	LGALS2	TSS1500	−0.33 (−0.44, −0.22)	3.98E-07	0.020
cg14422782	15	74312279	PML	Body	−0.16 (−0.21, −0.11)	4.66E-07	0.020
cg05596294	1	99729757	LPPR4	TSS200	−0.43 (−0.54, −0.31)	4.69E-07	0.020
cg07593390	3	136714183	IL20RB	Body	−0.41 (−0.55, −0.27)	4.82E-07	0.020
cg22136098	11	121297737			−0.19 (−0.25, −0.12)	4.90E-07	0.020
cg27210657	20	749360	C20orf54	TSS200	−0.34 (−0.43, −0.25)	5.17E-07	0.020
cg10086654	21	38339495	HLCS	TSS1500;5'UTR	−0.44 (−0.56, −0.33)	5.77E-07	0.020
cg26551913	10	102508286	PAX2	Body	−0.36 (−0.48, −0.24)	5.83E-07	0.020
cg22842539	3	131121232			−0.36 (−0.48, −0.24)	6.02E-07	0.020
cg25330005	4	90819638	MMRN1	Body	−0.34 (−0.45, −0.23)	6.02E-07	0.020
cg13939216	4	1888962	WHSC1	5'UTR	−0.31 (−0.41, −0.20)	6.31E-07	0.020

cg03466717	16	49564370	ZNF423	Body	−0.33 (−0.44, −0.22)	6.84E-07	0.021
cg26112909	1	22150263	LDLRAD2; HSPG2	3'UTR; Body	−0.51 (−0.69, −0.34)	7.50E-07	0.021
cg07394161	18	11179065			−0.36 (−0.49, −0.24)	7.65E-07	0.021
cg08839164	2	129494877			−0.28 (−0.38, −0.18)	7.66E-07	0.021
cg02214623	5	180671896	GNB2L1	TSS1500	−0.26 (−0.35, −0.17)	8.04E-07	0.021
cg11219895	19	17579559			−0.36 (−0.48, −0.23)	8.49E-07	0.021
cg17852032	6	158066811	ZDHHC14	Body	−0.27 (−0.37, −0.18)	8.90E-07	0.022
cg25729068	14	92303235	TC2N	TSS1500	−0.51 (−0.69, −0.33)	9.86E-07	0.022
cg14788563	6	20417986	E2F3	Body	−0.45 (−0.57, −0.33)	9.87E-07	0.022
cg16548911	16	50347766	ADCY7	Body	−0.37 (−0.50, −0.24)	1.24E-06	0.027
cg20691617	2	85142974			−0.14 (−0.20, −0.09)	1.30E-06	0.028
cg14321618	13	95989562			−0.41 (−0.56, −0.27)	1.43E-06	0.030
cg06404571	16	69167351	CHTF8	TSS1500	0.19 (0.12, 0.26)	1.53E-06	0.031
cg15347519	20	46912835			−1.28 (−1.66, −0.89)	1.62E-06	0.031
cg03957547	16	9524433			−1.57 (−2.06, −1.09)	1.73E-06	0.031
cg17164356	6	160209539	TCP1	5'UTR;Body	0.29 (0.19, 0.40)	1.74E-06	0.031
cg17091893	17	37756091			−0.16 (−0.21, −0.10)	1.74E-06	0.031
cg02049210	8	48647583	KIAA0146	Body	−0.34 (−0.46, −0.22)	1.81E-06	0.031
cg05990080	3	37965818	CTDSPL	Body	−0.24 (−0.33, −0.16)	1.82E-06	0.031
cg02366262	11	121297716			−0.21 (−0.29, −0.14)	1.96E-06	0.033
cg27493649	6	30042987	RNF39	Body	−0.32 (−0.44, −0.21)	2.05E-06	0.034
cg09543627	11	3228622			0.41 (0.27, 0.56)	2.11E-06	0.034
cg06811094	1	200272112			−0.67 (−0.87, −0.46)	2.20E-06	0.035
cg04972979	20	749148	C20orf54	1stExon;5'UTR	−0.31 (−0.41, −0.22)	2.35E-06	0.035
cg08904008	8	39806299	IDO2	Body	−0.52 (−0.71, −0.33)	2.42E-06	0.035

cg06179222	1	3410984	MEGF6	Body	−0.16 (−0.21, −0.12)	2.47E-06	0.035
cg15141868	7	37959966	EPDR1	TSS200	−0.35 (−0.48, −0.22)	2.47E-06	0.035
cg19340837	6	163647753	PACRG	Body	0.49 (0.31, 0.67)	2.60E-06	0.035
cg22681084	22	30476829	HORMAD2	5'UTR	−0.46 (−0.63, −0.29)	2.63E-06	0.035
cg01524661	19	2263663			−0.26 (−0.36, −0.17)	2.63E-06	0.035
cg02216981	2	175190675	LINC01305	TSS200	−0.25 (−0.34, −0.16)	2.66E-06	0.035
cg01383955	22	42828447	NFAM1	TSS200	−0.45 (−0.61, −0.28)	2.75E-06	0.035
cg25134852	2	71701721	DYSF	Body	−0.34 (−0.46, −0.21)	2.76E-06	0.035
cg21528155	1	61598558	NFIA	Body	−0.43 (−0.59, −0.27)	2.79E-06	0.035
cg05818894	11	27740078	BDNF	Body;5'UTR	−0.36 (−0.49, −0.23)	2.86E-06	0.035
cg01586330	9	140067764	TMEM210	TSS1500	0.18 (0.11, 0.25)	2.90E-06	0.035
cg26726768	3	136477443			−0.38 (−0.52, −0.24)	2.90E-06	0.035
cg23928744	1	110765802	KCNC4	Body	−0.30 (−0.40, −0.19)	2.92E-06	0.035
cg14995797	8	57232871	SDR16C5	1stExon;5'UTR	−0.27 (−0.35, −0.19)	3.02E-06	0.035
cg00565790	12	116985884			−0.32 (−0.43, −0.20)	3.04E-06	0.035
cg18322631	13	111634669	LINC00431	Body	0.44 (0.28, 0.60)	3.12E-06	0.035
cg16968662	1	897308	KLHL17	Body	−0.16 (−0.22, −0.10)	3.21E-06	0.035
cg00830604	15	39992499	FSIP1	Body	0.55 (0.38, 0.72)	3.21E-06	0.035
cg23528791	6	154799663	CNKSR3	Body	−0.21 (−0.29, −0.13)	3.24E-06	0.035
cg14070323	14	101291856	MEG3	TSS1500	−0.09 (−0.12, −0.06)	3.35E-06	0.036
cg07941017	5	179498303	RNF130	Body	−0.50 (−0.69, −0.32)	3.51E-06	0.037
cg07780377	11	77939490	GAB2	Body	−0.27 (−0.38, −0.17)	3.57E-06	0.037
cg14316450	13	46190134	ERICH6B	TSS1500	−0.33 (−0.45, −0.21)	3.65E-06	0.037
cg14972330	3	60003816	FHIT	Body	0.37 (0.23, 0.51)	3.70E-06	0.037
cg23692219	14	104571156	ASPG	Body	−0.34 (−0.45, −0.24)	3.79E-06	0.037

cg10142237	22	38714395	CSNK1E	TSS1500	−0.21 (−0.29, −0.13)	3.81E-06	0.037
cg04982488	10	116103698	AFAP1L2	Body	−0.42 (−0.58, −0.27)	3.86E-06	0.037
cg18529246	5	154027481			−0.41 (−0.53, −0.29)	3.89E-06	0.037
cg19590421	14	51327604			0.34 (0.21, 0.47)	4.07E-06	0.038
cg08135406	3	23785035			−0.49 (−0.68, −0.31)	4.18E-06	0.038
cg22051146	5	177895047	COL23A1	Body	−0.36 (−0.50, −0.23)	4.18E-06	0.038
cg15718164	3	17364661	TBC1D5	Body	−0.70 (−0.96, −0.44)	4.21E-06	0.038
cg08865365	19	45948021			−0.24 (−0.32, −0.15)	4.33E-06	0.038
cg05015022	14	52019598	FRMD6	5'UTR	−0.24 (−0.32, −0.17)	4.37E-06	0.038
cg13177873	20	17661522	RRBP1	5'UTR	−0.22 (−0.30, −0.14)	4.40E-06	0.038
cg08436616	21	29765894			−0.39 (−0.53, −0.24)	4.41E-06	0.038
cg15837205	12	68100230			−0.22 (−0.30, −0.14)	4.42E-06	0.038
cg04993628	2	232428244			−0.34 (−0.45, −0.24)	4.52E-06	0.038
cg02871960	6	11747325	ADTRP	Body	0.45 (0.28, 0.62)	4.61E-06	0.038
cg24671153	6	32140799	AGPAT1	5'UTR	−0.20 (−0.28, −0.13)	4.65E-06	0.038
cg27597392	12	12753718			−0.25 (−0.35, −0.16)	4.78E-06	0.039
cg08489732	15	66923544	LINC01169	Body	−0.19 (−0.26, −0.12)	4.82E-06	0.039
cg18985828	2	467799			−0.18 (−0.24, −0.13)	4.95E-06	0.039
cg19754901	3	87102643			0.50 (0.31, 0.69)	4.99E-06	0.039
cg08558340	7	100472263	SRRT	TSS1500	0.54 (0.37, 0.71)	4.99E-06	0.039
cg14617979	5	171590078	STK10	Body	−0.17 (−0.22, −0.12)	5.05E-06	0.039
cg21333338	17	2808456	RAP1GAP2	Body	−0.27 (−0.37, −0.17)	5.17E-06	0.040
cg00160780	11	124611639	NRGN	Body	−0.16 (−0.21, −0.10)	5.26E-06	0.040
cg08361238	1	117452304	PTGFRN	TSS1500	−0.22 (−0.30, −0.14)	5.37E-06	0.040
cg16692249	14	21268482			−0.37 (−0.51, −0.23)	5.44E-06	0.040

cg00539347	22	30592296			−0.38 (−0.53, −0.24)	5.47E-06	0.040
cg02264818	13	96072921			−0.43 (−0.59, −0.26)	5.55E-06	0.040
cg18344466	19	57702324	ZNF264	TSS1500	−0.52 (−0.69, −0.35)	5.64E-06	0.041
cg07850987	17	46645972	HOXB3	5'UTR	−0.31 (−0.43, −0.19)	5.72E-06	0.041
cg26033526	6	32819858	TAP1	Body	0.38 (0.23, 0.52)	5.86E-06	0.041
cg27066321	1	10534234	PEX14	TSS1500	−0.51 (−0.71, −0.32)	5.93E-06	0.041
cg03670892	15	75532245			−0.24 (−0.33, −0.15)	6.15E-06	0.042
cg23096476	21	44497077	CBS	TSS1500	−0.44 (−0.58, −0.30)	6.18E-06	0.042
cg02004851	5	140207460	PCDHA2	Body	−0.37 (−0.48, −0.25)	6.19E-06	0.042
cg07852557	17	79422586	BAHCC1	Body	−0.21 (−0.29, −0.13)	6.21E-06	0.042
cg13094752	14	23305548	MMP14	TSS1500	−0.19 (−0.26, −0.12)	6.39E-06	0.042
cg25499646	19	1811720	ATP8B3	5'UTR; Body	−0.21 (−0.28, −0.14)	6.45E-06	0.042
cg11304950	4	15703603	BST1	TSS1500	−0.35 (−0.49, −0.22)	6.51E-06	0.042
cg10110288	5	55274030	IL6ST	Body;5'UTR	0.25 (0.15, 0.34)	6.51E-06	0.042
cg10236224	16	30689953			−0.27 (−0.37, −0.17)	6.53E-06	0.042
cg15498349	19	39574712	PAPL	TSS1500	−0.38 (−0.53, −0.24)	6.55E-06	0.042
cg18788040	3	128952552			−0.26 (−0.37, −0.16)	6.59E-06	0.042
cg19622451	10	100227419	HPSE2	Body	−0.55 (−0.76, −0.34)	6.73E-06	0.042
cg04797575	4	176709392	GPM6A	Body	0.61 (0.37, 0.84)	6.89E-06	0.043
cg07061765	11	70349076	SHANK2	Body	−0.20 (−0.27, −0.12)	7.06E-06	0.043
cg01715248	9	93953608			−0.25 (−0.34, −0.15)	7.25E-06	0.043
cg02445972	17	7166593	CLDN7	TSS1500	−0.25 (−0.35, −0.15)	7.29E-06	0.043
cg25568061	2	71291953			−0.29 (−0.40, −0.18)	7.30E-06	0.043
cg27206367	18	76398311			0.38 (0.24, 0.53)	7.34E-06	0.043
cg26441055	1	42995495			−0.53 (−0.70, −0.37)	7.37E-06	0.043

cg12368137	4	153831263	ARFIP1	Body	−0.47 (−0.65, −0.29)	7.38E-06	0.043
cg02706658	13	33607403	KL	Body	−0.59 (−0.78, −0.40)	7.52E-06	0.044
cg25882580	19	54506534	CACNG6	Body	0.44 (0.27, 0.61)	7.59E-06	0.044
cg01441777	22	38714416	CSNK1E	TSS1500	−0.19 (−0.27, −0.12)	7.67E-06	0.044
cg25344795	16	68551729			−0.22 (−0.30, −0.13)	7.87E-06	0.044
cg04033732	16	67707096			−0.17 (−0.23, −0.10)	7.90E-06	0.044
cg23289456	20	749620	C20orf54	TSS1500	−0.39 (−0.52, −0.26)	7.93E-06	0.044
cg13417776	5	94992843	RFESD; SPATA9	3'UTR; Body	0.67 (0.41, 0.93)	8.06E-06	0.044
cg04826180	17	4938748	SLC52A1	TSS200	−0.29 (−0.40, −0.17)	8.06E-06	0.044
cg22481770	3	156324118			−0.32 (−0.43, −0.22)	8.09E-06	0.044
cg03159660	11	2078197			0.38 (0.26, 0.51)	8.20E-06	0.044
cg26842801	5	55070008	DDX4	Body	0.72 (0.49, 0.96)	8.38E-06	0.044
cg02471915	22	39040634	LOC646851	Body	0.22 (0.14, 0.31)	8.40E-06	0.044
cg09643443	18	32239802	DTNA	5'UTR	−0.34 (−0.47, −0.21)	8.42E-06	0.044
cg24921030	2	139192329			−0.26 (−0.36, −0.16)	8.47E-06	0.044
cg11962082	6	119418027	FAM184A	5'UTR	−0.30 (−0.40, −0.21)	8.47E-06	0.044
cg17183752	1	3508884	MEGF6	Body	0.52 (0.32, 0.73)	8.57E-06	0.044
cg10066188	2	73927710	NAT8B	1stExon;3'UTR	−0.30 (−0.41, −0.18)	8.58E-06	0.044
cg26926516	18	10851591	PIEZO2	Body	−0.50 (−0.69, −0.30)	8.61E-06	0.044
cg01868616	11	75041600	ARRB1	Body	−0.27 (−0.37, −0.16)	8.87E-06	0.045
cg13271643	13	21036583	CRYL1	Body	−0.22 (−0.31, −0.14)	8.98E-06	0.045
cg09508450	11	76389551			−0.19 (−0.27, −0.12)	9.01E-06	0.045
cg22953960	10	22766081			−0.41 (−0.54, −0.27)	9.03E-06	0.045
cg15972821	11	121192423			−0.19 (−0.26, −0.11)	9.04E-06	0.045
cg24975688	21	46786707			0.27 (0.17, 0.38)	9.15E-06	0.045

cg25972578	10	92548822	HTR7	Body	−0.36 (−0.49, −0.22)	9.19E-06	0.045
cg21827912	4	86763803	ARHGAP24	Body;5'UTR	−0.47 (−0.66, −0.29)	9.28E-06	0.045
cg26014511	8	22079390	PHYHIP	Body	0.42 (0.25, 0.58)	9.43E-06	0.045
cg21539594	1	43765867	TIE1	TSS1500	−0.27 (−0.38, −0.16)	9.48E-06	0.045
cg23202167	19	42442230			−0.26 (−0.37, −0.16)	9.58E-06	0.045
cg02930866	3	15482580	EAF1	3'UTR	−0.33 (−0.46, −0.20)	9.61E-06	0.045
cg21309469	19	18548153	ISYNA1	Body	0.81 (0.49, 1.13)	9.71E-06	0.046
cg01974027	1	114694766	SYT6	5'UTR	−0.39 (−0.54, −0.24)	9.82E-06	0.046
cg06709331	7	100881367	CLDN15	5'UTR	−0.16 (−0.22, −0.09)	1.01E-05	0.047
cg13348156	8	21991816			−0.26 (−0.37, −0.16)	1.04E-05	0.048
cg20311502	16	80705636	CDYL2	Body	−0.40 (−0.56, −0.24)	1.05E-05	0.048
cg17500342	14	77382482			−0.39 (−0.54, −0.23)	1.05E-05	0.048
cg09996840	17	79680718	SLC25A10	Body	−0.34 (−0.45, −0.23)	1.06E-05	0.048
cg07050213	2	11898882	LPIN1	Body	−0.35 (−0.49, −0.21)	1.08E-05	0.048
cg21160344	22	35752708			−0.40 (−0.56, −0.24)	1.08E-05	0.048
cg25317338	2	133669525	MIR7853; NCKAP5	Body	0.35 (0.24, 0.47)	1.09E-05	0.048
cg11960980	20	39874156	ZHX3	5'UTR	−0.16 (−0.22, −0.09)	1.09E-05	0.048
cg17141215	3	12791166	TMEM40	Body	−0.28 (−0.39, −0.17)	1.10E-05	0.048
cg14565651	1	9145034	SLC2A5	Body	−0.24 (−0.33, −0.14)	1.11E-05	0.048
cg06583200	4	76824629	PPEF2	TSS1500	0.55 (0.33, 0.77)	1.13E-05	0.049
cg10256708	19	18262564	PIK3R2	TSS1500	−0.21 (−0.30, −0.13)	1.13E-05	0.049
cg25301532	20	43378953	KCNK15	Body	0.60 (0.43, 0.77)	1.17E-05	0.050
cg08782920	11	31835884	PAX6	5'UTR	−0.22 (−0.30, −0.13)	1.17E-05	0.050
cg08500112	5	59086432	PDE4D	Body	−0.27 (−0.38, −0.17)	1.17E-05	0.050
cg07951201	13	103340215	C13orf39	Body	−0.37 (−0.52, −0.22)	1.19E-05	0.050

Note: Body refers to gene regions; TSS1500 denotes the region 1500 bp upstream of the transcription start site (TSS); 5'UTR indicates the 5' untranslated region; TSS200 represents the region 200 bp upstream of the transcription start site; 3'UTR signifies the 3' untranslated region; 1stExon pertains to the first exon region. CpG refers to cytosine－phosphate－guanine dinucleotides. The model was adjusted for gender, age, body mass index, occupation, education level, physical activity, frequency of drinking, particulate matter exposure, and leukocyte composition. Regression coefficients (β) represent the associations between vitamin D supplementation and DNA methylation M-values.

Table S8 Association between Vitamin D intervention-related CpGs and TLR4 concentrations (Top 20 ranked by *P*-value).

CpG	Chromosome	Gene	β (95% CI)	*P*-value	FDR adjust *P*-value
cg23096476	21	CBS	1.55 (1.01, 2.09)	7.24E-06	0.001
cg04982488	10	AFAP1L2	0.97 (0.56, 1.37)	2.64E-05	0.001
cg08558340	7	SRRT	−1.14 (−1.58, −0.69)	3.05E-05	0.001
cg27066321	1	PEX14	0.67 (0.36, 0.99)	1.48E-04	0.004
cg10086654	21	HLCS	1.20 (0.63, 1.76)	3.46E-04	0.008
cg25499646	19	ATP8B3	1.93 (0.93, 2.94)	4.82E-04	0.009
cg11962082	6	FAM184A	1.62 (0.75, 2.49)	9.65E-04	0.015
cg07593390	3	IL20RB	0.80 (0.32, 1.28)	2.14E-03	0.031
cg27210657	20	C20orf54	1.33 (0.53, 2.13)	2.44E-03	0.032
cg02706658	13	KL	0.61 (0.23, 1.00)	3.31E-03	0.038
cg00830604	15	FSIP1	−0.68 (−1.11, −0.24)	4.15E-03	0.045
cg10142237	22	CSNK1E	1.33 (0.46, 2.20)	4.44E-03	0.045
cg12202498	3	EAF1	0.86 (0.29, 1.42)	4.78E-03	0.046
cg09996840	17	SLC25A10	1.10 (0.36, 1.84)	6.06E-03	0.055
cg05990080	3	CTDSPL	1.13 (0.35, 1.91)	6.92E-03	0.058
cg07951201	13	C13orf39	0.71 (0.21, 1.21)	7.50E-03	0.058
cg09643443	18	DTNA	0.74 (0.22, 1.25)	7.85E-03	0.058
cg08361238	1	PTGFRN	1.17 (0.35, 1.99)	7.89E-03	0.058
cg01868616	11	ARRB1	0.90 (0.26, 1.54)	8.79E-03	0.060
cg11960980	20	ZHX3	1.58 (0.44, 2.72)	9.47E-03	0.060

The model was adjusted for gender, age, body mass index, occupation, education level, physical activity, frequency of drinking, particulate matter exposure, and leukocyte composition. Regression coefficients (β) represent the associations between DNA methylation M-values at individual CpG sites and log-transformed TLR4 concentrations.

Table S9 Association between Vitamin D intervention-related CpGs and ICAM-1 Concentrations (Top 20 ranked by *P*-value).

CpG	Chromosome	Gene	β (95% CI)	*P*-value	FDR adjust *P*-value
cg07593390	3	IL20RB	−0.54 (−0.91, −0.18)	5.69E-03	0.985
cg13417776	5	RFESD; SPATA9	0.23 (0.03, 0.43)	2.84E-02	1.000
cg04993628	2	-	−0.62 (−1.20, −0.05)	4.00E-02	1.000
cg14995797	8	SDR16C5	−0.70 (−1.49, 0.10)	9.47E-02	1.000
cg13939216	4	WHSC1	0.42 (−0.09, 0.92)	1.13E-01	1.000
cg25301532	20	KCNK15	−0.32 (−0.71, 0.07)	1.17E-01	1.000
cg06404571	16	CHTF8	0.62 (−0.15, 1.38)	1.22E-01	1.000
cg04797575	4	GPM6A	0.18 (−0.05, 0.41)	1.25E-01	1.000
cg21539594	1	TIE1	−0.39 (−0.87, 0.10)	1.27E-01	1.000
cg23096476	21	CBS	−0.43 (−1.00, 0.14)	1.45E-01	1.000
cg21309469	19	ISYNA1	−0.13 (−0.30, 0.04)	1.46E-01	1.000
cg16968662	1	KLHL17	0.69 (−0.26, 1.64)	1.61E-01	1.000
cg23928744	1	KCNC4	0.35 (−0.15, 0.84)	1.75E-01	1.000
cg02004851	5	PCDHA	0.36 (−0.16, 0.88)	1.79E-01	1.000
cg22051146	5	COL23A1	−0.25 (−0.63, 0.13)	2.01E-01	1.000
cg26112909	1	LDLRAD2; HSPG2	−0.20 (−0.50, 0.10)	2.01E-01	1.000
cg26551913	10	PAX2	−0.27 (−0.69, 0.15)	2.19E-01	1.000
cg27066321	1	PEX14	−0.17 (−0.45, 0.10)	2.27E-01	1.000
cg15498349	19	PAPL	0.21 (−0.13, 0.55)	2.32E-01	1.000
cg04826180	17	SLC52A1	−0.29 (−0.78, 0.20)	2.54E-01	1.000

The model was adjusted for gender, age, body mass index, occupation, education level, physical activity, frequency of drinking, particulate matter exposure, and leukocyte composition. Regression coefficients (β) represent the associations between DNA methylation M-values at individual CpG sites and log-transformed ICAM-1 concentrations.

Table S10 Association between Vitamin D intervention-related CpGs and IL-6 Concentrations (Top 20 ranked by *P*-value).

CpG	Chromosome	Gene	β (95% CI)	*P*-value	FDR adjust *P*-value
cg11962082	6	FAM184A	1.38 (0.87, 1.90)	1.75E-05	0.003
cg23096476	21	CBS	0.97 (0.57, 1.38)	4.69E-05	0.004
cg08500112	5	PDE4D	0.80 (0.40, 1.20)	3.51E-04	0.015
cg11304950	4	BST1	0.52 (0.26, 0.78)	4.46E-04	0.015
cg09996840	17	SLC25A10	0.89 (0.42, 1.36)	5.50E-04	0.016
cg25317338	2	MIR7853; NCKAP5	−0.90 (−1.40, −0.41)	8.28E-04	0.016
cg05990080	3	CTDSPL	0.91 (0.41, 1.40)	8.32E-04	0.016
cg17164356	6	TCP1	−0.74 (−1.14, −0.33)	8.99E-04	0.016
cg14995797	8	SDR16C5	1.12 (0.49, 1.76)	1.23E-03	0.019
cg05015022	14	FRMD6	1.01 (0.43, 1.59)	1.41E-03	0.020
cg10086654	21	HLCS	0.65 (0.26, 1.04)	2.33E-03	0.031
cg14070323	14	MEG3	1.99 (0.72, 3.26)	4.02E-03	0.043
cg26014511	8	PHYHIP	−0.42 (−0.69, −0.14)	5.02E-03	0.051
cg18344466	19	ZNF264	0.55 (0.18, 0.92)	5.46E-03	0.051
cg00129557	13	KL	0.29 (0.10, 0.49)	5.67E-03	0.051
cg07593390	3	IL20RB	0.47 (0.15, 0.79)	5.94E-03	0.051
cg14788563	6	E2F3	0.52 (0.15, 0.90)	8.34E-03	0.066
cg26551913	10	PAX2	0.47 (0.13, 0.81)	1.08E-02	0.079
cg25301532	20	KCNK15	-0.44 (-0.76, -0.12)	1.11E-02	0.079
cg22681084	22	HORMAD2	0.35 (0.09, 0.62)	1.17E-02	0.079

The model was adjusted for gender, age, body mass index, occupation, education level, physical activity, frequency of drinking, particulate matter exposure, and leukocyte composition. Regression coefficients (β) represent the associations between DNA methylation M-values at individual CpG sites and log-transformed IL-6 concentrations.

Table S11 Association between Vitamin D intervention-related CpGs and TNF-α concentrations (Top 20 ranked by *P*-value).

CpG	Chromosome	Gene	β (95% CI)	*P*-value	FDR adjust *P*-value
cg25882580	19	CACNG6	−0.61 (−0.90, −0.33)	1.44E-04	0.015
cg06709331	7	CLDN15	1.56 (0.78, 2.34)	2.89E-04	0.015
cg23528791	6	CNKSR3	1.18 (0.58, 1.79)	4.02E-04	0.015
cg00830604	15	FSIP1	−0.58 (−0.88, −0.28)	5.18E-04	0.015
cg17141215	3	TMEM40	0.82 (0.38, 1.25)	6.13E-04	0.015
cg14565651	1	SLC2A5	0.97 (0.45, 1.49)	7.14E-04	0.015
cg12210036	1	GOLT1A	0.70 (0.32, 1.08)	7.26E-04	0.015
cg27493649	6	RNF39	0.78 (0.36, 1.21)	7.70E-04	0.015
cg10142237	22	CSNK1E	1.12 (0.50, 1.75)	1.03E-03	0.016
cg25499646	19	ATP8B3	1.27 (0.53, 2.01)	1.54E-03	0.021
cg01868616	11	ARRB1	0.82 (0.34, 1.30)	1.59E-03	0.021
cg10066188	2	NAT8B	0.74 (0.30, 1.18)	1.81E-03	0.022
cg07593390	3	IL20RB	0.59 (0.24, 0.95)	2.01E-03	0.023
cg08361238	1	PTGFRN	0.98 (0.37, 1.59)	2.95E-03	0.027
cg18344466	19	ZNF264	0.59 (0.24, 0.95)	3.02E-03	0.027
cg07852557	17	BAHCC1	1.00 (0.37, 1.62)	3.11E-03	0.027
cg04969406	22	LGALS2	0.71 (0.26, 1.16)	3.16E-03	0.027
cg06404571	16	CHTF8	−1.14 (−1.86, −0.41)	3.71E-03	0.027
cg26014511	8	PHYHIP	−0.50 (−0.82, −0.18)	3.84E-03	0.027
cg03466717	16	ZNF423	0.67 (0.23, 1.11)	4.33E-03	0.027

The model was adjusted for gender, age, body mass index, occupation, education level, physical activity, frequency of drinking, particulate matter exposure, and leukocyte composition. Regression coefficients (β) represent the associations between DNA methylation M-values at individual CpG sites and log-transformed TNF-α concentrations.

Table S12 Association between Vitamin D intervention-related CpGs and IFNα2 concentrations (Top 20 ranked by *P*-value).

CpG	Chromosome	Gene	β (95% CI)	*P*-value	FDR adjust *P*-value
cg13094752	14	MMP14	−0.84 (−1.37, −0.30)	4.28E-03	0.515
cg10110288	5	IL6ST	0.58 (0.18, 0.98)	7.16E-03	0.515
cg07951201	13	C13orf39	−0.38 (−0.67, −0.09)	1.39E-02	0.515
cg25330005	4	MMRN1	−0.44 (−0.80, −0.09)	1.86E-02	0.515
cg26842801	5	DDX4	0.34 (0.05, 0.63)	2.92E-02	0.515
cg17141215	3	TMEM40	−0.40 (−0.75, −0.05)	3.23E-02	0.515
cg16548911	16	ADCY7	−0.35 (−0.68, −0.03)	3.92E-02	0.515
cg02706658	13	KL	−0.26 (−0.51, −0.01)	4.64E-02	0.515
cg03466717	16	ZNF423	−0.39 (−0.75, −0.02)	4.66E-02	0.515
cg15141868	7	EPDR1	−0.35 (−0.68, −0.01)	4.70E-02	0.515
cg25882580	19	CACNG6	0.26 (0.01, 0.51)	4.98E-02	0.515
cg02004851	5	PCDHA	−0.42 (−0.82, −0.01)	5.01E-02	0.515
cg02049210	8	KIAA0146	−0.35 (−0.70, 0.00)	5.86E-02	0.515
cg07780377	11	GAB2	−0.40 (−0.80, 0.00)	5.93E-02	0.515
cg20311502	16	CDYL2	−0.26 (−0.53, 0.01)	6.26E-02	0.515
cg00160780	11	NRGN	−0.68 (−1.40, 0.03)	6.80E-02	0.524
cg06709331	7	CLDN15	−0.64 (−1.32, 0.03)	6.97E-02	0.524
cg02445972	17	CLDN7	−0.41 (−0.86, 0.04)	8.11E-02	0.561
cg02216981	2	LINC01305	−0.40 (−0.87, 0.06)	9.53E-02	0.568
cg07941017	5	RNF130	−0.19 (−0.41, 0.03)	1.04E-01	0.599

The model was adjusted for gender, age, body mass index, occupation, education level, physical activity, frequency of drinking, particulate matter exposure, and leukocyte composition. Regression coefficients (β) represent the associations between DNA methylation M-values at individual CpG sites and log-transformed IFNα2 concentrations.
